# Testing the agreement of trees with internal labels

**DOI:** 10.1186/s13015-021-00201-9

**Published:** 2021-12-04

**Authors:** David Fernández-Baca, Lei Liu

**Affiliations:** Department of Computer Science, Iowa State University, Ames, IA USA

**Keywords:** Phylogenetic tree, Taxonomy, Agreement, Algorithm

## Abstract

****Background**:**

A semi-labeled tree is a tree where all leaves as well as, possibly, some internal nodes are labeled with taxa. Semi-labeled trees encompass ordinary phylogenetic trees and taxonomies. Suppose we are given a collection ${\mathcal {P}}= \{{\mathcal {T}}_1, {\mathcal {T}}_2, \ldots , {\mathcal {T}}_k\}$ of semi-labeled trees, called input trees, over partially overlapping sets of taxa. The agreement problem asks whether there exists a tree ${\mathcal {T}}$, called an agreement tree, whose taxon set is the union of the taxon sets of the input trees such that the restriction of ${\mathcal {T}}$ to the taxon set of ${\mathcal {T}}_i$ is isomorphic to ${\mathcal {T}}_i$, for each $i \in \{1, 2, \ldots , k\}$. The agreement problems is a special case of the supertree problem, the problem of synthesizing a collection of phylogenetic trees with partially overlapping taxon sets into a single supertree that represents the information in the input trees. An obstacle to building large phylogenetic supertrees is the limited amount of taxonomic overlap among the phylogenetic studies from which the input trees are obtained. Incorporating taxonomies into supertree analyses can alleviate this issue.

**Results:**

We give a ${\mathcal {O}}(n k (\sum _{i \in [k]} d_i + \log ^2(nk)))$ algorithm for the agreement problem, where *n* is the total number of distinct taxa in ${\mathcal {P}}$, *k* is the number of trees in ${\mathcal {P}}$, and $d_i$ is the maximum number of children of a node in ${\mathcal {T}}_i$.

**Conclusion:**

Our algorithm can aid in integrating taxonomies into supertree analyses. Our computational experience with the algorithm suggests that its performance in practice is much better than its worst-case bound indicates.

## Background

In the *agreement problem*, we are given a collection ${\mathcal {P}}= \{{\mathcal {T}}_1, {\mathcal {T}}_2, \ldots , {\mathcal {T}}_k\}$ of rooted phylogenetic trees with partially overlapping taxon sets. ${\mathcal {P}}$ is called a *profile* and the trees in ${\mathcal {P}}$ are the *input trees*. The question is whether there exists a tree ${\mathcal {T}}$ whose taxon set is the union of the taxon sets of the input trees such that ${\mathcal {T}}_i$ is isomorphic to the restriction of ${\mathcal {T}}$ to the taxon set of ${\mathcal {T}}_i$, for each $i \in \{1, 2, \ldots , k\}$. If such a tree ${\mathcal {T}}$ exists, then we call ${\mathcal {T}}$ an *agreement tree* for ${\mathcal {P}}$ and say that ${\mathcal {P}}$ *agrees*; otherwise, ${\mathcal {P}}$ *disagrees*.

Here we study a generalization of the agreement problem, where the internal nodes of the input trees may also be labeled. These labels represent higher-order taxa; that is, the labels stand for sets of taxa that may nest within each other. Thus, for example, an input tree may contain the taxon *Glycine max* (soybean) nested within a subtree whose root is labeled Fabaceae (the legumes), containing several other taxa, such as *Pisum sativum* (pea) and *Medicago sativa* (alfalfa). The Fabaceae subtree might itself be nested within a subtree whose root is labeled Angiospermae (flowering plants). Note that leaves themselves may be labeled by higher-order taxa. For example, the Fabaceae subtree may contain a leaf labeled *Phaseolus*, representing the bean genus. *Taxonomies* are examples of internally labeled trees. A taxonomy groups organisms according to a system of taxonomic rank (e.g., family, genus, and species). Two well-known taxonomies are the NCBI taxonomy [23] and the Angiosperm taxonomy [25].

We present a ${\mathcal {O}}(n k (\sum _{i \in [k]} d_i + \log ^2(nk)))$ algorithm for the agreement problem for trees with internal labels, where *n* is the total number of distinct taxa in ${\mathcal {P}}$, *k* is the number of trees in ${\mathcal {P}}$, and, for each $i \in \{1, 2, \dots , k\}$, $d_i$ is the maximum number of children of a node in ${\mathcal {T}}_i$. Our algorithm outputs an agreement supertree for the input trees if such a tree exists; if there is no agreement supertree, the algorithms reports this fact and terminates.

### Previous work

Ng and Wormald [18] gave the first explicit polynomial-time algorithm for the agreement problem for ordinary rooted phylogenetic trees (i.e., trees without internal labels)^1^. To our knowledge, the fastest algorithm for this problem runs in $O(n^2 k)$ time, where *n* is the number of distinct taxa in ${\mathcal {P}}$ [11].

The aforementioned algorithms are indebted to Aho et al.’s $\textsc {Build}$ algorithm [1], a relative of the agreement problem, the *compatibility problem*. The input to the compatibility problem is a profile ${\mathcal {P}}= \{{\mathcal {T}}_1, {\mathcal {T}}_2, \ldots , {\mathcal {T}}_k\}$ of rooted phylogenetic trees with partially overlapping taxon sets. The question is whether there exists a tree ${\mathcal {T}}$ whose taxon set is the union of the taxon sets of the input trees such that each input tree ${\mathcal {T}}_i$ can be obtained from the restriction of ${\mathcal {T}}$ to the taxon set of ${\mathcal {T}}_i$ through edge contractions. If such a tree ${\mathcal {T}}$ exists, we refer to ${\mathcal {T}}$ as a *compatible tree* for ${\mathcal {P}}$ and say that ${\mathcal {P}}$ is *compatible*; otherwise, ${\mathcal {P}}$ is *incompatible*.

Compatibility is a less stringent requirement than agreement: any profile that agrees is compatible, but the converse is not true. The compatibility problem for ordinary phylogenetic trees is solvable in ${\mathcal {O}}(M_{\mathcal {P}}\log ^2 M_{\mathcal {P}})$ time, where $M_{\mathcal {P}}$ is the total number of nodes and edges in the trees of ${\mathcal {P}}$ [10]. Note that $M_{\mathcal {P}}= {\mathcal {O}}(nk)$.

Compatibility and agreement reflect two distinct approaches to dealing with *multifurcations*; i.e., non-binary nodes, also known as *polytomies*. Suppose that node *v* is a multifurcation in some input tree of ${\mathcal {P}}$ and that $\ell _1$, $\ell _2$, and $\ell _3$ are taxa in three distinct subtrees of *v*. In an agreement tree for ${\mathcal {P}}$, these three taxa must be in distinct subtrees of some node in the agreement tree. In contrast, a compatible tree for ${\mathcal {P}}$ may contain no such node, since a compatible tree is allowed to “refine” the multifurcation at *v*—that is, group two out of $\ell _1$, $\ell _2$, and $\ell _3$ separately from the third. Thus, compatibility treats multifurcations as “soft” facts; agreement treats them as “hard” facts [17]. Both viewpoints can be valid, depending on the circumstances.

The need for agreement trees to respect the multifurcations in the input trees appears to make testing for agreement harder than testing for compatibility. Indeed, to handle agreement, a costly re-merging step must be added to $\textsc {Build}$. In this step, certain sets of the taxon partition generated by $\textsc {Build}$ are re-combined to reflect multifurcations [11, 18]. Similar issues are faced when testing consistency of triples and fans [16]. The situation is more complex for internally labeled trees, because internal nodes with the same label, but in different trees, may jointly imply multifurcations, even if all input trees are binary.

The agreement and compatibility problems are fundamental special cases of the *supertree problem*, the problem of synthesizing a collection of phylogenetic trees with partially overlapping taxon sets into a single supertree that represents the information in the input trees [2, 5, 20, 26]. The original supertree methods were limited to input trees where only the leaves are labeled (that is, ordinary phylogenetic trees), but there has been increasing interest in incorporating internally labeled trees in supertree analysis, motivated by the desire to incorporate taxonomies in these analyses. Taxonomies provide structure and completeness that can be hard to obtain otherwise [14, 19, 21], offering a way to circumvent one of the obstacles to building comprehensive phylogenies: the limited taxonomic overlap among different phylogenetic studies [22].

Although internally labeled trees, and taxonomies in particular, are not, strictly speaking, phylogenies, they have many of the same mathematical properties as phylogenies. Both phylogenies and internally labeled trees are *X*-*trees* (also called *semi-labeled trees*) [6, 24]. Nevertheless, algorithmic results for compatibility and agreement of internally labeled trees are scarce, compared to those for ordinary phylogenies. To our knowledge, the first algorithm for testing compatibility of internally labeled trees is in [8] (see also [4]). The fastest known algorithm for the problem runs in ${\mathcal {O}}(M_{\mathcal {P}}\log ^2 M_{\mathcal {P}})$ time [9]. We are unaware of any previous algorithms for the agreement problem for internally labeled trees.

### Organization of the paper

In the next section (“Preliminaries”), we provide formal definitions of rooted *X*-trees and agreement, as well as a characterization of agreement in terms of lowest common ancestors. We also introduce the display graph, which has a central role in our agreement algorithm. The subsequent section (“Decomposing a Profile”) studies the decomposability properties of profiles that agree. These properties allow us to reduce an agreement problem on a profile into independent agreement problems on subprofiles, leading to the agreement algorithm presented in the section titled “Constructing an agreement subtree”. We report our computational experiences with an implementation of our algorithm in the section titled “Experiments”.

### Note

This paper is an extended version of conference paper [13]. The present version contains proofs and has a new section describing our computational experience with an implementation of our algorithm.

## Preliminaries

For each positive integer *r*, [*r*] denotes the set $\{1, \dots , r\}$.

### Graphs and trees

Let *G* be a graph. *V*(*G*) and *E*(*G*) denote the node and edge sets of *G*. Let *U* be a subset of *V*(*G*). The *subgraph of* *G* *induced by* *U* is the graph whose vertex set is *U* and whose edge set consists of all of the edges in *E*(*G*) that have both endpoints in *U*. We write $G \setminus U$ to denote the graph obtained by deleting from *G* the nodes in *U*, along with their incident edges.

Let *u* and *v* be two nodes in *V*(*G*). Node *v* is *reachable* from *u* if there exists a path from *u* to *v*. The *connected components* of *G* are the equivalence classes of nodes under the “is reachable from” relation. Let *U* and *W* be two subsets of *V*(*G*). We say that *U* and *W* are *disconnected* if no node in *W* is reachable from a node in *U*.

A *tree* is an acyclic connected graph. All trees here are assumed to be rooted. For a tree *T*, *r*(*T*) denotes the root of *T*. Suppose $u, v \in V(T)$. Then, *u* is an *ancestor* of *v* in *T*, denoted $u \le _T v$, if *u* lies on the path from *v* to *r*(*T*) in *T*. If $u \le _T v$, then *v* is a *descendant* of *u*. Node *u* is a *proper ancestor* of *v*, denoted $u <_T v$, if $u \le _T v$ and $u\ne v$. We write $u \parallel _T v$ if neither $u \le _T v$ nor $v \le _T u$. If $\{u,v\} \in E(T)$ and $u \le _T v$, then *u* is the *parent* of *v* and *v* is a *child* of *u*.

Consider any $x \in V(T)$. We write $\text{parent}_T(x)$ and ${\text {Ch}}_T(x)$ to denote the parent of *x* and the set of children of *x*, respectively. The *subtree of* *T* *rooted at* *x*, denoted *T*(*x*), is the subtree of *T* consisting of all $y \in V(T)$ such that $x \le _T y$. We say that node *x* is a *multifurcation* if $|{\text {Ch}}_T(x)| > 2$.

We extend the child notation to subsets of *V*(*T*) in the natural way: for $U \subseteq V(T)$, ${\text {Ch}}_T(U) = \bigcup _{u \in U} {\text {Ch}}_T(u)$. Thus, if $U = \emptyset$, ${\text {Ch}}_T(U) = \emptyset$.

Let *T* be a tree and suppose $U \subseteq V(T)$. The *lowest common ancestor of* *U* *in* *T*, denoted $\text {LCA}_T(U)$, is the unique smallest upper bound of *U* under $\le _T$. A node $x \in U$ is a *minimal node of* *T* *in* *U* if for all $y \in U$, either $x \parallel _T y$ or $x \le _T y$. Note that a set *U* may have multiple minimal nodes and that for any $x \in V(T)$, $x = \text {LCA}_T(V(T(x))$ and *x* is the unique minimal node of *T*(*x*) in *V*(*T*(*x*)).

### Rooted *X*-trees

Throughout the paper, *X* denotes a set of *labels* (that is, taxa, which may be, for instance, species or families of species). A *rooted* *X*-*tree* (or *X*-tree, for short), also known as a *semi-labeled tree*, is a pair ${\mathcal {T}}= (T,\phi )$ where *T* is a rooted tree and $\phi$ is a mapping from *X* to *V*(*T*) such that, for every node $v \in V(T)$ with at most one child, $v \in \phi (X)$. *X* is the *label set* of ${\mathcal {T}}$ and $\phi$ is the *labeling function* of ${\mathcal {T}}$. For every node $v \in V(T)$, $\phi ^{-1}(v)$ denotes the (possibly empty) subset of *X* whose elements map into *v*; these elements as the *labels of* *v*. If $\phi ^{-1}(v) \ne \emptyset$, then *v* is *labeled*; otherwise, *v* is *unlabeled*. For $U \subseteq V(T)$, we write $\phi ^{-1}(U)$ to denote $\bigcup _{u \in U} \phi ^{-1}(u)$.

By definition, every leaf in an *X*-tree is labeled, and any node, including the root, that has a single child must be labeled. Nodes with two or more children may be labeled or unlabeled. An *X*-tree ${\mathcal {T}}= (T,\phi )$ is *singularly labeled* if every node in *T* has at most one label; ${\mathcal {T}}$ is *fully labeled* if every node in *T* is labeled.

*X*-trees generalize ordinary phylogenetic trees (also known as *phylogenetic* *X-trees* [24]). An ordinary phylogenetic tree is a semi-labeled tree ${\mathcal {T}}= (T,\phi )$ where *r*(*T*) has at least two children and $\phi$ is a bijection from *X* into leaf set of *T* (thus, internal nodes are not labeled).

Let ${\mathcal {T}}= (T,\phi )$ be an *X*-tree. For each $u \in V(T)$, *X*(*u*) denotes the set of all labels in *T*(*u*); that is, $X(u) = \bigcup _{v: u \le _T v} \phi ^{-1}(v)$. *X*(*u*) is called a *cluster* of *T*. ${\text {Cl}}({\mathcal {T}})$ denotes the set of all clusters of ${\mathcal {T}}$. We extend the cluster notation to sets of nodes as follows. Let *U* be a subset of *V*(*T*). Then, $X(U) = \bigcup _{v \in U} X(v)$. If $U = \emptyset$, then $X(U) = \emptyset$.

Suppose $Y \subseteq X$ for an *X*-tree ${\mathcal {T}}= (T,\phi )$. The *restriction* of ${\mathcal {T}}$ to *Y*, denoted ${\mathcal {T}}|Y$, is the semi-labeled tree whose cluster set is ${\text {Cl}}({\mathcal {T}}| Y) = \{W \cap Y : W \in {\text {Cl}}({\mathcal {T}}) \text { and } W \cap Y \ne \emptyset \}.$ Intuitively, ${\mathcal {T}}| Y$ is obtained from the minimal rooted subtree of *T* that connects the nodes in $\phi (Y)$ by suppressing all vertices *v* such that $v \notin \phi (Y)$ and *v* has only one child.

Let ${\mathcal {T}}= (T,\phi )$ be an *X*-tree and ${\mathcal {T}}' = (T', \phi ')$ be an $X'$-tree such that $X' \subseteq X$. ${\mathcal {T}}$ *agrees with* ${\mathcal {T}}'$ if ${\text {Cl}}({\mathcal {T}}') = {\text {Cl}}({\mathcal {T}}|X')$. It is well known that the clusters of a tree determine the tree, up to isomorphism [24, Theorem 3.5.2]. Thus, ${\mathcal {T}}$ agrees with ${\mathcal {T}}'$ if ${\mathcal {T}}'$ and ${\mathcal {T}}|X'$ are isomorphic.

### Profiles and agreement

**Fig. 1:**
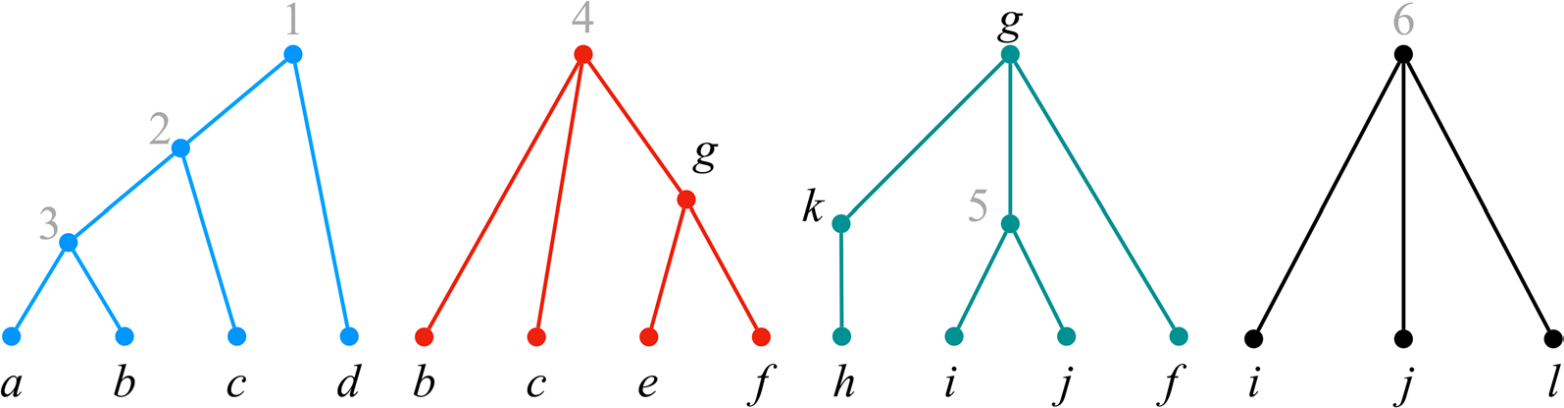
A profile ${\mathcal {P}}= \{{\mathcal {T}}_1, {\mathcal {T}}_2, {\mathcal {T}}_3, {\mathcal {T}}_4\}$. The letters are the original labels; grey numbers are labels added to make the trees fully labeled. We use this profile as a running example throughout the paper

Throughout the rest of this paper, ${\mathcal {P}}$ denotes a set $\{{\mathcal {T}}_1, {\mathcal {T}}_2, \dots , {\mathcal {T}}_k\}$ such that, for each $i \in [k]$, ${\mathcal {T}}_i = (T_i, \phi _i)$ is an $X_i$-tree for some label set $X_i$ (Fig. 1). We refer to ${\mathcal {P}}$ as a *profile*, and to the trees in ${\mathcal {P}}$ as *input trees*. We write $X_{\mathcal {P}}$ to denote $\bigcup _{i \in [k]} X_i$.

**Fig. 2:**
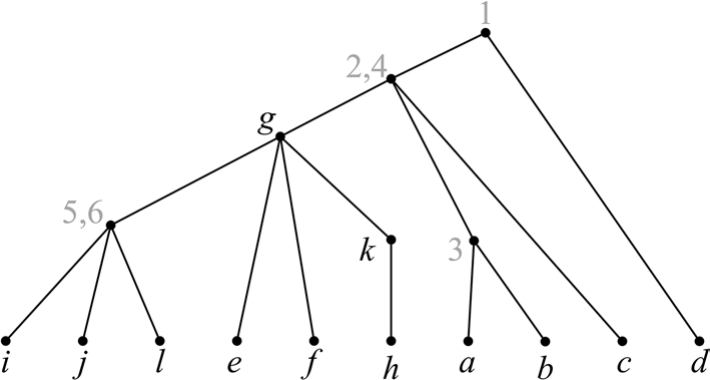
An agreement tree for ${\mathcal {P}}$. Although all input trees are singularly labeled, the agreement tree is not

A profile ${\mathcal {P}}$ *agrees* if there is an $X_{\mathcal {P}}$-tree ${\mathcal {T}}$ that agrees with each of the trees in ${\mathcal {P}}$. If ${\mathcal {T}}$ exists, we refer to ${\mathcal {T}}$ as an *agreement tree for* ${\mathcal {P}}$. See Fig. 2.

Given a subset *Y* of $X_{\mathcal {P}}$, the *restriction* of ${\mathcal {P}}$ to *Y*, denoted ${\mathcal {P}}|Y$, is the profile defined as ${\mathcal {P}}|Y = \{{\mathcal {T}}_1|Y \cap X_1, {\mathcal {T}}_2|Y \cap X_2, \dots , {\mathcal {T}}_k|Y \cap X_k\}.$

#### **Lemma 1**

*Suppose a profile *${\mathcal {P}}$ *has an agreement tree* ${\mathcal {T}}$*. Then, for any* $Y \subseteq X_{\mathcal {P}}$, ${\mathcal {T}}| Y$ *is an agreement tree for* ${\mathcal {P}}| Y$.

#### *Proof*

Consider any $i \in [k]$. Since ${\mathcal {T}}$ agrees with ${\mathcal {T}}_i$, $A \in {\text {Cl}}({\mathcal {T}}_i)$ if and only if $A \in {\text {Cl}}({\mathcal {T}}|X_i)$. Thus, for any $Y \subseteq X_{\mathcal {P}}$, if $Y_i = Y \cap X_i$, then $A \cap Y_i \in {\text {Cl}}({\mathcal {T}}_i|Y_i)$ if and only if $A \cap Y \in {\text {Cl}}({\mathcal {T}}|Y_i)$. The lemma follows. $\square$

We can convert a profile ${\mathcal {P}}$ containing trees that are not fully labeled into an equivalent profile ${\mathcal {P}}'$ of fully-labeled trees as follows. For each $i \in [k]$, let $l_i$ be the number of unlabeled nodes in $T_i$. Create a set $X'$ of $n' = \sum _{i \in [k]} l_i$ labels such that $X' \cap X_{\mathcal {P}}= \emptyset$. For each $i \in [k]$ and each $v \in V(T_i)$ such that $\phi _i^{-1}(v) = \emptyset$, make $\phi _i^{-1}(v) = \{\ell \}$, where $\ell$ is a distinct element from $X'$. We refer to ${\mathcal {P}}'$ as the *profile obtained by adding distinct new labels to* ${\mathcal {P}}$. See Fig. 1.

The proof of the next result is analogous to that of [8, Lemma 3.4].

#### **Lemma 2**

*Let *${\mathcal {P}}'$ *be the profile obtained by adding distinct new labels to *${\mathcal {P}}$*. Then, *${\mathcal {P}}$ *agrees if and only if *${\mathcal {P}}'$ *agrees. Further, if *${\mathcal {T}}$ *is an agreement tree for *${\mathcal {P}}'$*, then* ${\mathcal {T}}$ *is also an agreement tree for* ${\mathcal {P}}$.

#### *Proof*

Let ${\mathcal {P}}' = \{{\mathcal {T}}_1', {\mathcal {T}}_2', \dots , {\mathcal {T}}_k'\}$. For each $i \in [k]$, let $X'_i$ be the set of new labels added to ${\mathcal {T}}_i$ to obtain ${\mathcal {T}}_i'$. By definition, if ${\mathcal {T}}$ is an agreement tree for ${\mathcal {P}}'$, then, for each $i \in [k]$, $A \in {\text {Cl}}({\mathcal {T}}_i')$ if and only if $A \in {\text {Cl}}({\mathcal {T}}|(X_i \cup X_i'))$. To prove the lemma, it suffices now to show that, for each $i \in [k]$, $A \in {\text {Cl}}({\mathcal {T}}_i')$ if and only if $A \cap X_i \in {\text {Cl}}({\mathcal {T}}_i)$. We omit the details. $\square$

From this point forward, we make the following assumption.

#### **Assumption 1**

For each $i \in [k]$, ${\mathcal {T}}_i$ is fully and singularly labeled.

Lemma 2 implies that no generality is lost in assuming that all trees in ${\mathcal {P}}$ are fully labeled. Note that even if the trees in ${\mathcal {P}}$ are singularly labeled, a tree that agrees with ${\mathcal {P}}$ is not necessarily singularly labeled. See Fig. 2.

By Assumption 1, for each $i \in [k]$, there is a bijection between the labels in $X_i$ and the nodes of $V(T_i)$. (As noted earlier, however, if ${\mathcal {T}}= (T,\phi )$ is an agreement tree for ${\mathcal {P}}$, then $\phi$ is not in general a bijection between $X_{\mathcal {P}}$ and *V*(*T*).) For this reason, we will often refer to nodes of the input trees by their labels. In particular, given a label $\ell \in X_i$, we write $X_i(\ell )$ to denote $X_i(\phi _i(\ell ))$ (the cluster of ${\mathcal {T}}_i$ at the node labeled $\ell$), ${\text {Ch}}_{T_i}(\ell )$ to denote $\phi _i({\text {Ch}}_{T_i}(\phi _i(\ell ))$ (the labels of children of $\ell$ in ${\mathcal {T}}_i$), and ${\text {Ch}}_{T_i}(A)$ to denote $\phi _i^{-1}({\text {Ch}}_{T_i}(\phi _i(A))$, for $A \subseteq X_i$.

The following characterization of agreement generalizes a result in [11].

#### **Lemma 3**

*Let* ${\mathcal {P}}$ *be a profile and *${\mathcal {T}} = (T, \phi )$ *be an *$X_{\mathcal {P}}$*-tree. Then,* ${\mathcal {T}}$ *is an agreement tree for* ${\mathcal {P}}$ *if and only if for each* $i \in [k]$ *and each label *$a \in X_i$,

1. $$\phi (a) = \text {LCA}_T(X_i(a))$$,
2. for each label $b \in {\text {Ch}}_{T_i}(a)$, $\phi (a) <_T \phi (b)$, and
3. for every two distinct labels $b, c \in {\text {Ch}}_{T_i}(a)$, there exist distinct nodes $u, v \in {\text {Ch}}_T(\phi (a))$ such that $\phi (b) \in X_{\mathcal {P}}(u)$ and $\phi (c) \in X_{\mathcal {P}}(v)$.

#### *Proof*

(*If*) Suppose that $\phi$ satisfies conditions (E1)–(E3). To prove that ${\mathcal {T}}$ agrees with ${\mathcal {T}}_i$, we show that ${\text {Cl}}({\mathcal {T}}_i) = {\text {Cl}}({\mathcal {T}}|X_i)$.

First, we show that ${\text {Cl}}({\mathcal {T}}_i) \subseteq {\text {Cl}}({\mathcal {T}}|X_i)$ by arguing that $X_i(a) = X_{\mathcal {P}}(\phi (a)) \cap X_i$, for each $a \in X_i$. By (E1), $X_i(a) \subseteq X_{\mathcal {P}}(\phi (a))$. Now, suppose that there is a label $b \in X_{\mathcal {P}}(\phi (a)) \cap X_i$ such that $b \notin X_i(a)$. Let $c = \text {LCA}_{T_i}(X_i(a) \cup \{b\})$. Then, since $b \notin X_i(a)$, $X_i(a) \subset X_i(c)$. Hence, $c <_{T_i} a$ and, by (E2), $\phi (c) <_T \phi (a)$. Thus, (i) there exist distinct labels $d, d' \in {\text {Ch}}_{T_1}(c)$ such that $a \in X_i(d)$ and $b \in X_i(d')$. but (ii) since $a, b \in X_{{\mathcal {P}}}(\phi (a)) \cap X_i$, there is a single child *u* of ${\text {Ch}}_{{\mathcal {P}}}(c)$ such that $a, b \in X_{{\mathcal {P}}}(v)$, contradicting condition (E3).

Next, we prove that ${\text {Cl}}({\mathcal {T}}|X_i) \subseteq {\text {Cl}}({\mathcal {T}}_i)$. Suppose, to the contrary, that there is a cluster $Y \in {\text {Cl}}({\mathcal {T}}|X_i) \setminus {\text {Cl}}({\mathcal {T}}_i)$. Let $u = \text {LCA}_T(Y)$; thus, $Y = X_{\mathcal {P}}(u) \cap X_i$. Let $a = \text {LCA}_{T_i}(Y)$. Then, $Y \subset X_i(a)$. Choose any $b \in X_i(a) \setminus Y$; thus, $a \le _{T_i} b$. Note that $b \notin X_{\mathcal {P}}(u)$ and $\phi (a) <_T u$. We have two cases.

(i) $a \ne b$. Then, $a <_{T_i} b$. On the other hand, we have either $\phi (a) \ge _T \phi (b)$ or $\phi (a) \parallel _T \phi (b)$, contradicting (E2).
(ii) $a = b$. Then, there exist distinct labels $c_1, c_2 \in {\text {Ch}}_{T_i}(a)$ such that $Y \cap X_i(c_j) \ne \emptyset$ and $Y \not \subseteq X_i(c_j)$, for $j \in [2]$. By (E1), $\phi (a) = \text {LCA}_T(X_i(a))$. Since $\phi (a) <_T u$, there exists a unique node $v \in {\text {Ch}}_T(u)$ such that $Y \subset X_{\mathcal {P}}(v)$. But then $\phi (c_1)$ and $\phi (c_2)$ descend from the same child, *v*, of $\phi (a)$, contradicting condition (E3).

(*Only if*) Suppose that ${\mathcal {T}}$ agrees with ${\mathcal {T}}_i$. It is straightforward to show that $\phi$ must satisfy (E1). Thus, we focus on conditions (E2) and (E3).

Suppose condition (E2) does not hold. Then, there exists a label $b \in {\text {Ch}}_{T_i}(a)$, such that $\phi (a) \ge _{T} \phi (b)$. Since $X_{\mathcal {P}}(\phi (b)) \ne X_{\mathcal {P}}(\phi (a))$, we must have $\phi (a) >_{T} \phi (b)$. But then ${\mathcal {T}}$ does not agree with ${\mathcal {T}}_i$, a contradiction.

Suppose condition (E3) does not hold. Then, there exist distinct labels $c, c' \in {\text {Ch}}_{T_i}(a)$ such that $\{\phi (c), \phi (c')\} \subseteq X_{\mathcal {P}}(v)$, for some $v \in {\text {Ch}}_T(\phi (a))$. But then ${\mathcal {T}}|X_i$ contains cluster $Y = X_{\mathcal {P}}(v) \cap X_i$, which is not in ${\mathcal {T}}_i$, contradicting the assumption that ${\mathcal {T}}$ agrees with ${\mathcal {T}}_i$. $\square$

#### **Lemma 4**

*If profile *${\mathcal {P}}$ *agrees, then *${\mathcal {P}}$ *has an agreement tree* ${\mathcal {T}}= (T,\phi )$ *such that* $\phi ^{-1}(v) \ne \emptyset$ *for each node* $v \in V(T)$.

#### *Proof*

Suppose there is a node $v \in V(T)$ such that $\phi ^{-1}(v) = \emptyset$. Note that *v* cannot be a leaf. Let $u_1, u_2, \ldots , u_d$ be the children of *v*. We use the following fact.

**Fact.** *For each* $i \in [k]$, *there is at most one* $j \in [d]$ *such that* $X_{\mathcal {P}}(u_j) \cap X_i \ne \emptyset$.

**Proof**

Assume to the contrary that there exist distinct $j, j' \in [d]$ such that $W = X_{\mathcal {P}}(u_j) \cap X_i \ne \emptyset$ and $W' = X_{\mathcal {P}}(u_{j'}) \cap X_i \ne \emptyset$. Let $c = \text {LCA}_{T_i}(W)$ and $c' = \text {LCA}_{T_i}(W')$ and let $a = \text {LCA}_{T_i}(W \cup W')$. Then, *c* and $c'$ are in distinct subtrees of $T_i(a)$. By Lemma 3, $\phi (c)$ and $\phi (c')$ are in distinct subtrees of *v* and $v = \phi (a)$. But this contradicts the assumption that $\phi ^{-1}(v) = \emptyset$. $\Box$

Now, choose any $j \in [d]$. Let $T'$ be the tree obtained by contracting the edge $(v,u_j) \in E(T)$. That is, $T'$ is obtained by eliminating edge $(v,u_j)$, deleting $u_j$, and making ${\text {Ch}}_{T'}(v) = {\text {Ch}}_T(v) \cup {\text {Ch}}_T(u_j)$. Let ${\mathcal {T}}' = (T', \phi ')$, where $(\phi ')^{-1}(w) = \phi ^{-1}(w)$, if $w \in V(T) \setminus \{v,u_j\}$, and $(\phi ')^{-1}(v) = \phi ^{-1}(v) \cup \phi ^{-1}(u_j)$. Then, the above fact implies that, for each $i \in [k]$, ${\text {Cl}}({\mathcal {T}}|X_i) = {\text {Cl}}({\mathcal {T}}'|X_i)$. That is, ${\mathcal {T}}'$ is also an agreement tree for ${\mathcal {P}}$. Let ${\mathcal {T}}'' = (T'',\phi '')$ be the tree that results from repeating this contraction operation until it no longer applies. Then, ${\mathcal {T}}''$ satisfies $(\phi '')^{-1}(v) \ne \emptyset$ for each node $v \in V(T'')$. $\square$

### The display graph

**Fig. 3:**
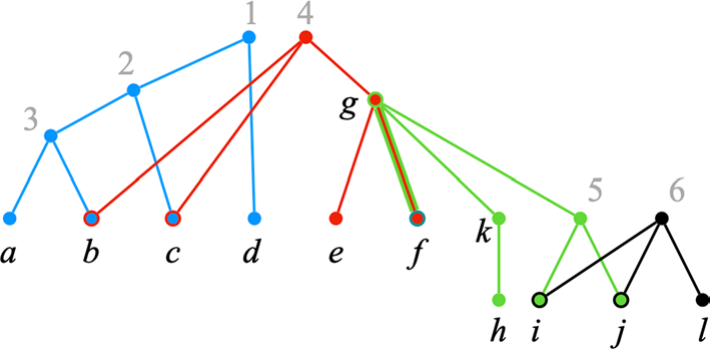
Display graph. The display graph $H_{\mathcal {P}}$ of the profile of Fig. 1

The *display graph* of a profile ${\mathcal {P}}$, denoted $H_{\mathcal {P}}$, is the graph obtained from the disjoint union of the underlying trees $T_1, \dots , T_k$ of ${\mathcal {P}}$ by identifying nodes that have the same label (parallel edges are replaced by a single edge) [7, 9, 10]. See Fig. 3. As we shall see, $H_{\mathcal {P}}$ plays a major role in our agreement algorithm.

$H_{\mathcal {P}}$ has *O*(*nk*) nodes and edges, and can be constructed in *O*(*nk*) time. By Assumption 1, there is a bijection between the labels in *X* and the nodes of $H_{\mathcal {P}}$. Thus, from this point forward, we refer to the nodes of $H_{\mathcal {P}}$ by their labels.

## Decomposing a profile

A *position* in a profile ${\mathcal {P}}$ is a tuple $\pi = (\pi _1, \pi _2, \dots , \pi _k)$ where $\pi _i \subseteq X_i$, for each $i \in [k]$. At any given point during its execution, our agreement algorithm focuses on testing the agreement of the subprofile of ${\mathcal {P}}$ determined by the subtrees associated with a specific position.

The *initial position* for ${\mathcal {P}}$ is the position $\pi ^\text {init}$, where, for each $i \in [k]$, $\pi ^\text {init}_i$ is a singleton set consisting of the label of $r(T_i)$; i.e., $\pi ^\text {init}_i = \phi _i^{-1}(r(T_i))$. In the profile of Fig. 1, $\pi ^\text {init}= (\{1\},\{4\},\{g\},\{6\})$.

Note that the definition of a position allows for the existence of $i, j \in [k]$, $i \ne j$, such that $\ell \in \pi _i$, but $\ell \notin \pi _{j}$, even if $\ell \in X_i$ and $\ell \in X_{j}$. Thus, for example, in the profile of Fig. 1, we have $g \in \pi ^\text {init}_3$, but $g \notin \pi ^\text {init}_2$, even though *g* appears in trees $T_3$ and $T_2$.

For a position $\pi$ in ${\mathcal {P}}$, let $X_{\mathcal {P}}(\pi )$ denote the set of labels $\bigcup _{i \in [k]} X_i(\pi _i)$. $H_{\mathcal {P}}(\pi )$ denotes the subgraph of $H_{\mathcal {P}}$ induced by $X_{\mathcal {P}}(\pi )$. Thus, $H_{\mathcal {P}}(\pi ^\text {init}) = H_{\mathcal {P}}$.

A position $\pi$ in ${\mathcal {P}}$ is *valid* if, for each $i \in [k]$,

1 $$\begin{aligned} \pi _i = {\left\{ \begin{array}{ll} \{\text {LCA}_{T_i}(X_i \cap X_{\mathcal {P}}(\pi )) \}, &{} \text {if}~{ X_i \cap X_{\mathcal {P}}(\pi ) \ne \emptyset ,} \\ \emptyset , &{} \text {otherwise} \end{array}\right. } \end{aligned}$$

Thus, if $\pi$ is valid, then, for each $i \in [k]$ such that $X_i \cap X_{\mathcal {P}}(\pi ) \ne \emptyset$, component $\pi _i$ consists of a single label $\ell$ such that $T_i(\ell )$ contains every label in $H_{\mathcal {P}}(\pi )$ that also belongs to $X_i$. Clearly, $\pi ^\text {init}$ is a valid position.

Let $\pi$ be a valid position. A label $\ell \in \bigcup _{i \in [k]} \pi _i$ is *exposed in* $\pi$ if $\pi _i = \{\ell \}$ for every $i \in [k]$ such that $\ell \in X_i \cap X_{\mathcal {P}}(\pi )$. A set $S \subseteq \bigcup _{i \in [k]} \pi _i$ is an *exposed subset in* $\pi$ (*exposed subset* for short, when $\pi$ is understood) if every label $\ell \in S$ is exposed.

Consider the initial position $\pi ^\text {init}$ of the profile of Fig. 1. Label 1 is exposed in $\pi ^\text {init}$ since $\pi ^\text {init}_1 = \{1\}$ and label 1 exists only in ${\mathcal {T}}_1$. Similarly, labels 4 and 6 are both exposed. On the other hand, label *g* is not exposed, since it appears in trees $T_2$ and $T_3$, but $g \notin \pi ^\text {init}_2$, even though $\pi ^\text {init}_3 = \{g\}$.

We say that a position $\pi$ *has an agreement tree* if ${\mathcal {P}}|X_{\mathcal {P}}(\pi )$ has an agreement tree.

### **Lemma 5**

*A profile *${\mathcal {P}}$ *has an agreement tree if and only if there exists an agreement tree for every valid position *$\pi$ *in* ${\mathcal {P}}$.

### *Proof*

(*Only if*) Suppose ${\mathcal {P}}$ has an agreement tree ${\mathcal {T}}$. For any valid position $\pi$ in ${\mathcal {P}}$, $X_{\mathcal {P}}(\pi ) \subseteq X_{\mathcal {P}}$. Thus, by Lemma 1, ${\mathcal {T}}|X_{\mathcal {P}}(\pi )$ is an agreement tree for $\pi$.

(*If*) Suppose there is an agreement tree for every valid position $\pi$ in ${\mathcal {P}}$. Then, in particular, there exists an agreement tree ${\mathcal {T}}$ for the initial position $\pi ^\text {init}$ of ${\mathcal {P}}$. Since $X_{\mathcal {P}}(\pi ^\text {init}) = X_{\mathcal {P}}$, ${\mathcal {T}}$ must also be an agreement tree for ${\mathcal {P}}$. $\square$

### Decomposing a position

In what follows, $\pi$ denotes a valid position in ${\mathcal {P}}$. For each $i \in [k]$ such that $\pi _i \ne \emptyset$, $\ell _i \in X_i$ denotes the single label in $\pi _i$.

Let *S* be an exposed subset of $\pi$. We say that *S* is *nice* if for each connected component *W* of $H_{\mathcal {P}}(\pi ) \setminus S$, the position $\pi ^{W}= (\pi _1^{W}, \pi _1^{W}, \dots , \pi _k^{W})$ defined as follows is valid:

2 $$\begin{aligned} \pi _i^{W} = \{a : a \hbox { is a minimal label of} T_i \hbox {in} \, X_i \cap W\}. \end{aligned}$$

Observe that if *S* is nice, $W = X_{\mathcal {P}}(\pi ^W),$ for each connected component *W* of $H_{\mathcal {P}}(\pi ) \setminus S.$

If *S* is a nice exposed set, we refer to the set $\{\pi ^{W} : W \hbox { is a connected component of}\ H_{\mathcal {P}}(\pi ) \setminus S\}$ of valid positions as the *successor positions* of $\pi$ (with respect to *S*).

A *good decomposition* of $\pi$ is a pair $(S, \Pi )$, where *S* is a nice exposed subset and $\Pi$ is the collection of successor positions of $\pi$ with respect to $\pi$. Note that $X_{\mathcal {P}}(\pi ) = S \cup \bigcup _{\pi ' \in \Pi } X_{\mathcal {P}}(\pi ')$. Note also that we allow *S* or $\Pi$ to be empty.

**Fig. 4:**
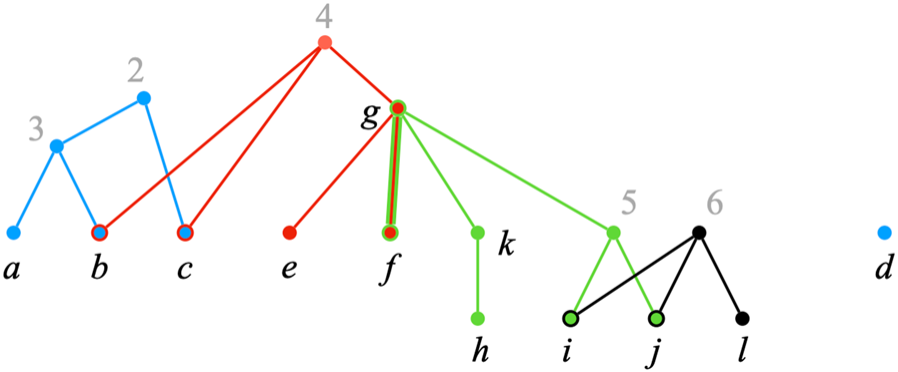
A decomposition of $\pi ^\text {init}$. $H_{\mathcal {P}}\setminus \{1\}$. Each connected component corresponds to a distinct successor position of $\pi ^\text {init}$

Consider the profile of Fig. 1, whose display graph is in Fig.  3. Let $S = \{1\}$. Figure 4 shows $H_{\mathcal {P}}(\pi ^\text {init}) \setminus S$. $H_{\mathcal {P}}(\pi ) \setminus S$ has two connected components, $W_1 =\{d\}$ and $W_2 = \{2,3,4,5,6,a,b,c,e,f,g,h, i,j,k,l\}$. Then, by Eq. (2), the corresponding positions are

$$\begin{aligned} \pi ^{W_1} = (\{d\},\emptyset ,\emptyset ,\emptyset ) \quad \text {and} \quad \pi ^{W_2} = (\{2\},\{4\},\{g\},\{6\}). \end{aligned}$$

Positions $\pi ^{W_1}$ and $\pi ^{W_2}$ are clearly valid positions. Therefore, *S* is nice and $(S, \Pi )$, where $\Pi = \{\pi ^{W_1}, \pi ^{W_2}\}$, is a good decomposition of $\pi ^\text {init}$.

The next result is central to our agreement algorithm.

#### **Lemma 6**

*Let* $\pi$ *be a valid position in a profile* ${\mathcal {P}}$*. Then,* $\pi$ *has an agreement tree if and only if there exists a good decomposition* $(S, \Pi )$ *of* $\pi$ *such that* $S \ne \emptyset$ *and, for each position* $\pi ' \in \Pi$, $\pi '$ *has an agreement tree. If such a good decomposition exists, then* $\pi$ *has an agreement tree* ${\mathcal {T}}= (T, \phi )$ *where* $\phi ^{-1}(r(T)) = S$.

#### *Proof*

(*Only if*) Suppose position $\pi$ has an agreement tree ${\mathcal {T}}= (T, \phi )$ (thus, ${\mathcal {T}}$ is an $X_{\mathcal {P}}(\pi )$-tree). Let $S = \phi ^{-1}(r(T))$. By Lemma 4, we can assume that $S \ne \emptyset$. Note that every label $\ell \in S$ must be in $\bigcup _{i \in [k]} \pi _i$. Further, $\ell$ must be exposed in $\pi$. Indeed, if $\ell$ is not exposed, there exists an $i \in [k]$ such that $\pi _i \ne \{\ell '\}$, where $\ell ' <_{T_i} \ell$, so $\ell \notin \phi ^{-1}(r(T))$, a contradiction.

If *T* consists of a single node $u = r(T)$, then we must have $S = \bigcup _{i \in [k]} \pi _i = X_{\mathcal {P}}(\pi )$. Then, $(S, \emptyset )$ is trivially a good decomposition of $\pi$.

Now, suppose ${\text {Ch}}_T(r(T)) = \{v_1, v_2,\dots , v_d\}$, where $d \ge 1$. For each $j \in [d]$, let ${\mathcal {T}}^{(j)} = {\mathcal {T}}|X_{\mathcal {P}}(v_j)$. By Lemma 1, ${\mathcal {T}}^{(j)}$ is an agreement tree for ${\mathcal {P}}|X_{\mathcal {P}}(v_j)$. For each $i \in [k]$ and each $j \in [d]$ such that $X_i \cap X_{\mathcal {P}}(v_j) \ne \emptyset$, let

$$\begin{aligned} \ell ^{(j)}_i = \text {LCA}_{T_i}(X_i \cap X_{\mathcal {P}}(v_j)). \end{aligned}$$

Thus, $\ell ^{(j)}_i$ is the root of ${\mathcal {T}}_i|\left( X_i \cap X_{\mathcal {P}}(v_j)\right)$.

For each $j \in [d]$, define a position $\pi ^{(j)}$, where, for each $i \in [k]$,

$$\begin{aligned} \pi ^{(j)}_i = {\left\{ \begin{array}{ll} \left\{ \ell ^{(j)}_i \right\} &{} \hbox { if}\ X_i \cap X_{\mathcal {P}}(v_j) \ne \emptyset \\ \emptyset &{} \text {otherwise.} \end{array}\right. } \end{aligned}$$

Let $\Pi = \{\pi ^{(1)}, \pi ^{(2)}, \dots , \pi ^{(d)}\}$. By construction, for each $j \in [d]$, $\pi ^{(j)}$ satisfies Eq. (1), so $\pi ^{(j)}$ is valid. Since $X_{\mathcal {P}}(v_j) = X_{\mathcal {P}}(\pi ^{(j)})$, ${\mathcal {T}}^{(j)}$ is an agreement tree for $\pi ^{(j)}$.

For any $j' \in [d]$ such that $j' \ne j,$ $X_{\mathcal {P}}(\pi ^{(j)})$ and $X_{\mathcal {P}}(\pi ^{(j')})$ are disconnected in $H_{\mathcal {P}}(\pi ) \setminus S,$ since every path between the two sets must go through a label in *S*. Note, however, that $X_{\mathcal {P}}(\pi ^{(j)})$ may contain multiple connected components of $H_{\mathcal {P}}(\pi ) \setminus S$. For each connected component *W* of $H_{\mathcal {P}}(\pi ^{(j)})$, let ${\mathcal {T}}^{(j,W)} = {\mathcal {T}}^{(j)} | W$ and let $\pi ^{(j,W)}$ be the position where $\pi ^{(j,W)}_i = \pi ^{(j)}_i \cap W$, for each $i \in [k]$. Then, ${\mathcal {T}}^{(j,W)}$ is an agreement tree for $\pi ^{(j,W)}.$

Let $\Pi$ consist of all positions $\pi ^{(j,W)}$ such that $j \in [d]$ and *W* is a connected component of $H_{\mathcal {P}}(\pi ^{(j)})$. Then $(S,\Pi )$ is a good decomposition of $\pi$, where each position in $\Pi$ has an agreement tree.

(*If*) Let $(S, \Pi )$ be a good decomposition of $\pi$ such that $S \ne \emptyset$ and each position in $\Pi$ has an agreement tree. If $\Pi = \emptyset$, then we must have $S = X_{\mathcal {P}}(\pi )$. Further, for each $i \in [k]$ such that $\pi _i \ne \emptyset$, it must be the case that $T_i$ consists of a single node, labeled by the single label in $\pi _i$. Let *T* be the tree consisting of a single node $u = r(T)$ and let $\phi (\ell ) = u$, for all $u \in S$. Then, ${\mathcal {T}}= (T,\phi )$ is an agreement tree for $\pi$.

Now suppose $\Pi \ne \emptyset$. Let $\Pi = \{\pi ^{(1)}, \pi ^{(2)}, \dots , \pi ^{(d)}\}$. For each $j \in [d]$, let ${\mathcal {T}}^{(j)} = (T^{(j)}, \phi ^{(j)})$ be an agreement tree for $\pi ^{(j)}$, and let $v_j$ be the root of $T^{(j)}$. Let ${\mathcal {T}}= (T, \phi )$ be the $X_{\mathcal {P}}(\pi )$-tree where *T* is assembled by creating a new node *u* and making ${\text {Ch}}_T(u) = \{v_1, v_2, \dots , v_d\}$ and, for each $\ell \in X_{\mathcal {P}}(\pi )$, $\phi (\ell )$ is defined as

$$\begin{aligned} \phi (\ell ) = {\left\{ \begin{array}{ll} u &{} \hbox { if}\ \ell \in S \\ \phi ^{(j)}(\ell ) &{} \text {if}~{ \ell \in X_{\mathcal {P}}(\pi ^{(j)}).} \end{array}\right. } \end{aligned}$$

Since $(S,\Pi )$ is a good decomposition, $S \cup \bigcup _{j \in [k]} X_{\mathcal {P}}(\pi ^{(j)}) = X_{\mathcal {P}}(\pi )$. Thus, ${\mathcal {T}}$ is an $X_{\mathcal {P}}(\pi )$-tree. We prove that ${\mathcal {T}}$ is an agreement tree for $\pi$, by showing that, for each $i \in [k]$, $\phi$ satisfies properties (E1)–(E3) of Lemma 3.

By Lemma 3 every label in ${\mathcal {T}}_i|X_i(\pi _i^{(j)})$ satisfies (E1)–(E3). For each $j \in [d]$, let $\ell _i$ be the label of the root of ${\mathcal {T}}_i|X_i(\pi _i^{(j)})$. There are two possibilities:

(i) $\ell _i \in \phi ^{-1}(u)$. Then, each of $\ell _i$’s children must be in a distinct subtree of *u*. Thus, properties (E1)–(E3) are satisfied.
(ii) $\ell _i \not \in \phi ^{-1}(u)$. Then, $\ell _i$ and all of its children must be contained in a single subtree, say ${\mathcal {T}}_j$, of *u*, and the claim follows from the fact that $\phi ^{(j)}$ satisfies properties (E1)–(E3).

$$\square$$

### Good partitions

To find a good decomposition of a position $\pi$, it is convenient to work with partitions of the set of children of the labels in $\pi$. We write ${\text {Ch}}_{\mathcal {P}}(\pi )$ to denote the set of all children of some label in $\pi$; i.e., ${\text {Ch}}_{\mathcal {P}}(\pi ) = \bigcup _{i \in [k]} {\text {Ch}}_{T_i}(\pi _i)$.

Let *S* be an exposed subset of $\pi$. The *partition of* ${\text {Ch}}_{\mathcal {P}}(\pi )$ *induced by* *S*, denoted $\Psi (S)$, is the set consisting of all $A \subseteq {\text {Ch}}_{\mathcal {P}}(\pi )$ such that $A = {\text {Ch}}_{\mathcal {P}}(\pi ) \cap W$ for some connected component *W* of $H_{\mathcal {P}}(\pi ) \setminus S$

#### **Lemma 7**

*Let* *S be a subset of the exposed nodes in a valid position* $\pi$. *S* *is a nice set for *$\pi$ *if and only if for every set* $A \in \Psi (S)$ *and each* $a \in \cup _{i\in [k]} \pi _i$ *the following holds for all *$i \in [k]$ *such that *${\text {Ch}}_{T_i}(a) \cap A \ne \emptyset$.

1. If $$a \in S$$, then $$|{\text {Ch}}_{T_i}(a) \cap A | =1$$.
2. If $$a \notin S$$, then $${\text {Ch}}_{T_i}(a) \subseteq A$$.

#### *Proof*

Consider any $A \in \Psi (S)$. Let *W* be the connected component of $H_{\mathcal {P}}(\pi ) \setminus S$ containing *A* and let $\pi ^W$ be the position defined by Eq. (2). To prove the lemma, we show that $\pi ^W$ is valid if and only if conditions (N7) and (N7) hold.

($\Longrightarrow$) Suppose $\pi ^W$ is valid.

Consider any label $a \in S$ and any $i \in [k]$ such that ${\text {Ch}}_{T_i}(a) \cap A \ne \emptyset$. Since $\pi ^W$ is valid, $\pi _i^W = \{b\}$, where $b = \text {LCA}_{T_i}(X_i \cap W)$. Thus, *b* is a minimal label of $T_i$ in $X_i \cap W$, and so $b \in {\text {Ch}}_{T_i}(a)$. Thus $|{\text {Ch}}_{T_i}(a) \cap W| = 1$, and (N7) holds.

Consider any label $a \in \bigcup _{i \in [k]} \pi _i \setminus S$ such that ${\text {Ch}}_{T_i}(a) \cap A \ne \emptyset$. Then, every node in $T_i(a)$ must lie inside *W* and, since $\pi$ is valid, *a* is minimal in $X_i \cap W$. Thus, $\pi ^W_i = \pi _i$. Since *a* remains connected to all its children, ${\text {Ch}}_{T_i}(a) \subseteq A_j$, and thus (N7) holds.

($\Longleftarrow$) Suppose that for every $a \in \cup _{i\in [k]} \pi _i$ and every $i \in [k]$ such that ${\text {Ch}}_{T_i}(a) \cap A \ne \emptyset$, condition (N7) or (N7) holds, depending on whether or not $a \in S$.

Suppose $a \in S$. Consider any $i \in [k]$ such that ${\text {Ch}}_{T_i}(a) \cap A \ne \emptyset$. By (N7), *A* contains only one child $c \in {\text {Ch}}_{T_i}(a)$. We claim that $c = \text {LCA}_{T_i}(X_i \cap W)$. Assume, to the contrary that *W* contains another label $c'$ from $T_i(a)$, but $c' \notin V(T_i(c))$. By (N7), $c' \notin {\text {Ch}}_{T_i}(a)$. Suppose $c'$ is some descendant of another child *b* of *a*. But *b* must also be in *W*, contradicting (N7). Therefore, *c* is the minimal label of $T_i$ in $X_i \cap W$.

Suppose $a \in \bigcup _{i \in [k]} \pi _i \setminus S$. By condition (N7), $V(T_i(a))$ must be contained in *W*, and *a* is the minimal label of $T_i$ in $X_i \cap W$ because *a* is the root of $T_i(a)$.

Hence, $\pi ^W$ is valid, for each connected component *W* of $H_{\mathcal {P}}(\pi ) \setminus S$. Therefore, *S* is nice. $\square$

Suppose *S* is a nice exposed subset in a valid position $\pi$ and let *A* be any set in $\Psi (S)$. The *position associated with* *A* is the position $\pi ^A$, where, for each $i \in [k]$, $\pi _i^A$ is defined as follows. If $\pi _i = \emptyset$, then $\pi _i^A = \emptyset$. Otherwise, let *a* be the single element in $\pi _i$. Then,

3 $$\begin{aligned} \pi ^A_i = {\left\{ \begin{array}{ll} {\text {Ch}}_{T_i}(a) \cap A &{} \text {if }~{a \in S},~ \text {and} \\ \pi _i &{} \text {if}~{a \notin S.} \end{array}\right. } \end{aligned}$$

Consider the profile of Fig. 1, whose display graph is in Fig. 3. Note that

$$\begin{aligned} {\text {Ch}}_{\mathcal {P}}(\pi ^\text {init}) = \{2,d,b,c,g,k,5,f,i,j,l\}. \end{aligned}$$

Let $S = \{1\}.$ It can be verified that the partition $\Psi (S) = \{A,B\}$ of ${\text {Ch}}_{\mathcal {P}}(\pi ^\text {init})$ where

$$\begin{aligned} A = \{d\} \text { and } B = \{2,b,c,g,k,5,f,i,j,l\} \end{aligned}$$

satisfies the conditions of Lemma 7. Thus, *S* is a nice exposed subset. Using Eq. (3), we obtain

$$\begin{aligned} \pi ^{A} = (\{d\},\emptyset ,\emptyset ,\emptyset ) \text { and } \pi ^{B} = (\{2\},\{4\},\{g\},\{6\}). \end{aligned}$$

Observe that $(S,\Pi )$, where $\Pi = \{\pi ^A, \pi ^B\}$, is precisely the good decomposition of position $\pi ^\text {init}$ presented in the previous section. The next lemma shows that this is not a coincidence.

#### **Lemma 8**

*Suppose* *S* *is a nice exposed subset of *$\pi$.* Let *$\Pi = \{\pi ^A: A \in \Psi (S)\}$*. Then,* $(S,\Pi )$ *is a good decomposition of *$\pi$.

#### *Proof*

Let *A* be any set in $\Psi (S)$, *W* be the connected component of $H_{\mathcal {P}}(\pi ) \setminus S$ that contains *A*, and $\pi ^W$ be the position defined by Eq. (2). Since *S* is a nice set, $\pi ^W$ is valid. To prove the lemma it suffices to show that $\pi ^A = \pi ^W$. Consider each $i \in [k]$.

- If $\pi _i = \emptyset$, then we have $\pi ^A_i = \pi ^W_i = \emptyset$.
- Now, suppose $\pi _i = \{a\}$, for some $a \in \bigcup _{i \in [k]} \pi _i$.
  - If $a \in S$, then, by Lemma 7, ${\text {Ch}}_{T_i}(a) \cap A = \{c\}$, for some $c \in {\text {Ch}}_{T_i}(a)$. Then, $\pi ^A_i = \{c\}$. Since $a \notin W$, *c* must be the minimal label of $T_i$ in $X_i \cap W$. Therefore, $\pi ^A_i = \pi ^W_i = \{c\}$.
  - If $a \notin S$, then, by Lemma 7, ${\text {Ch}}_{T_i}(a) \subseteq A$. Then, $a \in W$ and, hence, *a* is the minimal label of $T_i$ in $X_i \cap W$. Therefore, $\pi ^A_i = \pi ^W_i$.

Thus, $\pi ^A = \pi ^W$ as claimed. $\square$

Motivated by Lemma 8, we say that a pair $(S, \Psi (S))$ is a *good partition* of ${\text {Ch}}_{\mathcal {P}}(\pi )$ if the pair $(S,\Pi )$ where $\Pi = \{\pi ^A: A \in \Psi (S)\}$ is a good decomposition of $\pi$.

### Maximal good decompositions

A valid position $\pi$ may have many possible nice exposed sets. We are interested in finding a *maximal* nice exposed subset; that is, a set *S* such that $S' \subseteq S$, for every nice exposed subset $S'$ of $\pi$.

#### **Lemma 9**

*Let* $\pi$ *be a valid position in a profile *${\mathcal {P}}$*. Then*, $\pi$ *has a unique maximal nice exposed subset.*

To prove Lemma 9, we need an auxiliary result.

#### **Lemma 10**

*Let* *S* *and* $S'$ *be two nice exposed subsets of *$\pi$*. Then* $S'' = S \cup S'$ *is also a nice exposed subset of* $\pi$.

#### *Proof*

Since *S* and $S'$ are exposed subsets, so is $S''$. By Lemma 7, the result follows from the next fact.

**Fact.** *Consider any set* $A \in \Psi (S'')$ *and any label* $a \in \bigcup _{i \in [k]} \pi _i$. *Then, for each* $i \in [k]$ *such that* ${\text {Ch}}_{T_i}(a) \cap A \ne \emptyset$, $|{\text {Ch}}_{T_i}(a) \cap A | =1$ *if* $a \in S''$* and* ${\text {Ch}}_{T_i}(a) \subseteq A$ *if* $a \notin S''$.

There are two cases to consider.

- Suppose $a \in S''$. Then, either $a \in S$ or $a \in S'$. Assume without loss of generality that $a \in S$. Set *A* is contained in some set in $B \in \Psi (S)$. By Lemma 7, $|{\text {Ch}}_{T_i}(a) \cap B| = 1$. Thus, $|{\text {Ch}}_{T_i}(a) \cap A| \le 1$. But ${\text {Ch}}_{T_i}(a) \cap A \ne \emptyset$, so $|{\text {Ch}}_{T_i}(a) \cap A| = 1$.
- Suppose $a \notin S''$. Since $a \notin S$ and $a \notin S'$ and ${\text {Ch}}_{T_i}(a) \cap A \ne \emptyset$, the connected component of $H_{\mathcal {P}}(\pi ) \setminus S''$ containing *A* contains *a* and thus ${\text {Ch}}_{T_i}(a) \subseteq A$.

$$\square$$

#### *Proof of Lemma 9*

Suppose, on the contrary, that there exist at least two distinct maximal nice exposed subsets $S, S'$. By Lemma 10, $S'' = S \cup S'$ is also a nice exposed subset of $\pi$. But $S \subset S''$, contradicting the maximality of *S*. $\square$

#### **Corollary 1**

*Let* $\pi$ *be a valid position in a profile *${\mathcal {P}}$ *and* *S* *be the maximal nice exposed subset of* $\pi$*. If* $\pi$ *has an agreement tree, then* $S \ne \emptyset$.

#### *Proof*

Suppose, on the contrary, that $\pi$ has an agreement tree, but $S = \emptyset$. Then, by Lemma 9, *every* nice exposed subset in $\pi$ must be empty. But, by Lemma 6, this implies that $\pi$ has no agreement tree, a contradiction. $\square$

Let *S* be the maximal nice exposed subset in $\pi$ and $\Pi$ be the set of successor positions of $\pi$ with respect to *S*. We refer to $(S,\Pi )$ as the *maximal good decomposition* of $\pi$.

## Constructing an agreement tree

Algorithm BuildAST(Algorithm 1) takes as input a profile ${\mathcal {P}}$ on a set of labels *X* and either returns an agreement tree for ${\mathcal {P}}$ or reports that no such tree exists. BuildAST assumes the availability of an algorithm Decompose, to be described later, that, given a valid position $\pi$ in ${\mathcal {P}}$, returns a maximal good decomposition $(S,\Pi )$ of $\pi$.

Algorithm 1: Testing agreement


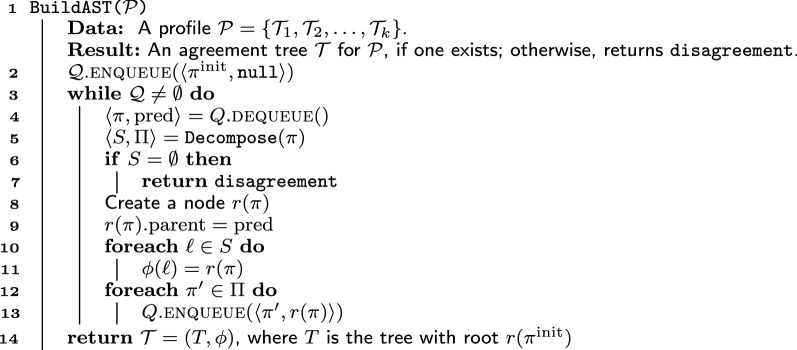


BuildAST proceeds from the top down, starting from the initial position $\pi ^\text {init}$ of ${\mathcal {P}}$, attempting to construct an agreement tree for ${\mathcal {P}}$ in a breadth-first manner. Like other algorithms based on breadth-first search, BuildAST uses a queue, which stores pairs $\langle \pi , \text{pred}\rangle$ where $\pi$ is a position in ${\mathcal {P}}$ and $\text{pred}$ is a reference to the parent of the tree node (potentially) to be created for $\pi$. At the outset, the queue contains only the pair $\langle \pi ^\text {init}, \mathtt {null}\rangle$, corresponding to the root of the agreement tree, which has no parent.

At each iteration of its outer **while** loop (lines 3–13), BuildAST extracts a pair $\langle \pi , \text{pred}\rangle$ from its queue and invokes Decompose to obtain a maximal good decomposition $(S,\Pi )$ of $\pi$. If $S = \emptyset$, then, by Corollary 1, no agreement tree for $\pi$ exists. BuildAST reports this fact (line 7) and terminates.

If $S \ne \emptyset$, BuildAST creates a tree node $r(\pi )$ for $\pi$; $r(\pi )$ is the tentative root for the agreement tree for $\pi$. By Lemma 6, if $\pi$ has an agreement subtree, then it has an agreement tree where $\phi (\ell ) = r(\pi )$. Lines 10–11 set up the mapping $\phi$ accordingly. Also by Lemma 6, if $\pi$ has an agreement tree, then so does each position $\pi ' \in \Pi$; furthermore, the roots of the trees for each position in $\Pi$ will be the children of $r(\pi )$. Thus, BuildAST adds $\langle \pi ', r(\pi ) \rangle$, for each $\pi ' \in \Pi$ to the queue, to ensure that $\pi '$ is processed at a later iteration and that the root of the agreement tree constructed for $\pi '$ (if such a tree exists) has $r(\pi )$ as its parent (lines 12–13). Therefore, if BuildAST terminates without reporting $\mathtt {disagreement}$, the result returned in line 14 is an agreement tree for ${\mathcal {P}}$. BuildAST indeed terminates, because there are only two possibilities at any given iteration: either the algorithm terminates reporting $\mathtt {disagreement}$ or (since $S \ne \emptyset$) the maximal good decomposition $(S, \Pi )$ of $\pi$ has the property that $\bigcup _{\pi ' \in \Pi } X_{\mathcal {P}}(\pi ')$ is a *proper* subset of $X_{\mathcal {P}}(\pi )$. The number of iterations of BuildAST cannot exceed the total number of nodes in an agreement tree for ${\mathcal {P}}$, which is *O*(*n*). Thus, we have the following result.

### **Theorem 1**

*Given a profile *${\mathcal {P}}= \{{\mathcal {T}}_1, {\mathcal {T}}_2, \dots , {\mathcal {T}}_k\}$, BuildAST *returns an agreement tree *${\mathcal {T}}$ *for* ${\mathcal {P}}$*, if such a tree exists; otherwise,* BuildAST *returns* $\mathtt {disagreement}$*. The total number of iterations of *BuildAST’*s outer loop is* *O*(*n*).

### Finding the maximal good decomposition

Algorithm 2: Computing the maximal good decomposition.


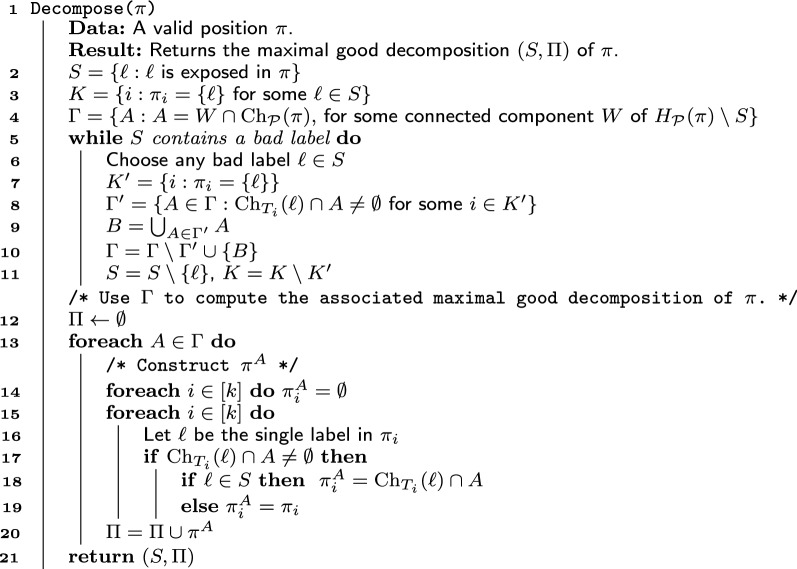


Algorithm Decompose (Algorithm 2) computes a maximal good decomposition of a position $\pi$. Throughout its execution, Decomposemaintains a set *S* that is a superset of the maximal nice exposed subset of $\pi$ and a partition $\Gamma$ of ${\text {Ch}}_{\mathcal {P}}(\pi )$. We will argue that before and after every iteration of the **while** loop of Lines  5–11, $\Gamma = \Psi (S)$. We will also show that, after the loop terminates, *S* is a maximal nice exposed subset. Lines 12–20 use *S* and $\Psi (S)$ to compute the maximal good decomposition of $\pi$. Next, we describe and analyze Decomposein detail.

Lines 2 and 3 of Decomposeinitialize *S* to contain all exposed labels in $\pi$ and *K* to consist of the indices of the trees in ${\mathcal {P}}$ that contain the labels in *S*. Line 11 initializes $\Gamma$ using $H_{\mathcal {P}}(\pi )$. We say that a label $\ell \in S$ is *bad* if there exist $i \in K$ and $A \in \Gamma$ such that $\pi _i = \{\ell \}$ and $|{\text {Ch}}_{T_i}(\ell ) \cap A| \ge 2$. Intuitively, a label $\ell$ is bad if $\ell$ must be a multifurcation in any agreement tree for ${\mathcal {P}}$, but at least two of $\ell$’s children lie in the same set in $\Gamma$, while the others lie in different sets.

Lines 5–11 of Decompose construct the maximal nice exposed subset by deleting bad labels from *S* and merging sets in $\Gamma$ accordingly. Conceptually, removing a bad label from *S* is equivalent to reinserting it into the graph. Thus, the union operations in the **while** loop of lines 5–11 can be interpreted as reconnecting bad labels to their children. In the implementation of Decompose, however, labels and the edges to their children are only deleted once. To understand why this is possible, observe that once a label $\ell$ becomes exposed in a position $\pi$, it remains exposed in every position where $\ell$ subsequently appears, until it is finally deleted from the graph or BuildASTterminates. Thus, conceptually, at every call to Decomposewhere $\ell$ is exposed, lines 2–11 add $\ell$ to *S* and delete $\ell$ from the graph, but then an iteration of lines 5–11 may possibly delete $\ell$ from *S* and reinsert it into the graph. Instead, our implementation of Decomposedeletes $\ell$ only once. When an iteration of lines  5–11 calls for deleting $\ell$ from *S*, instead of adding $\ell$ back to the graph, we put the various components that would have been reunited into a “virtual” connected component (a similar idea is used in [11]). We elaborate on our approach in the next section.

#### **Lemma 11**

*Let* $\pi$ *be a valid position in a profile* ${\mathcal {P}}$ *and let* $S^*$ *be the maximal nice exposed subset in* $\pi$*. Let* $S_j$ *and* $\Gamma _j$ *denote the values of* *S* *and* $\Gamma$ *after* *j iterations of the loop of Lines 5–11 of* Decompose, *and* *r denote the total number of iterations of the loop. Then,* $r \le k$, $\Gamma _j = \Psi (S_j)$*, for* $j \in \{0,1, \dots , r\}$*, and* $S_0 \supset S_1 \supset S_2 \supset \dots \supset S_r = S^*$.

#### *Proof*

The *j*th iteration of the loop, $j > 1$, removes one bad label from $S_{j-1}$. Thus, $S_j \subset S_{j-1}$. Since $|S| \le k$, the number of iterations is at most *k*.

Let us prove that $\Gamma _j = \Psi (S_j)$ and $S_j \supseteq S^*$, for each $j \in \{0,1, \dots , r\}$. $\Gamma _0 = \Psi (S_0)$ holds by construction and $S_0 \supseteq S^*$ holds trivially. Now assume that $\Gamma _{j-1} = \Psi (S_{j-1})$ and $S_{j-1} \supseteq S^*$. Note that $S_j = S_{j-1} \setminus \{\ell \}$, where $\ell$ is the bad label chosen in line 6. Since the body of the loop merges all the sets in $\Gamma _{j-1}$ that contain a child of $\ell$, we have $\Gamma _j = \Psi (S_j)$. Furthermore, $\ell$ cannot be in $S^*$, so $S_j \supseteq S^*$.

We claim that, for each $j \in \{0,1, \dots , r\}$, each $\ell \in \bigcup _{i \in [k]} \pi _i \setminus S_j$, there is an $A \in \Gamma _j$ such that ${\text {Ch}}_{\mathcal {P}}(\ell ) \subseteq X_{\mathcal {P}}(A)$. This is true by construction for $j = 0$, and the body of the **while** loop ensures that this remains true throughout the execution of the algorithm.

At termination of the **while** loop, $S_r$ contains no bad labels. Thus, $\Gamma _r = \Psi (S_r)$ satisfies the conditions of Lemma 7 with respect to $S_r$. Thus, $(S,\Psi (S_r))$ is a good partition of ${\text {Ch}}_{\mathcal {P}}(\pi )$.

When the loop of lines  5–11 terminates, $S_r$ is a maximal nice exposed subset in $\pi$. By Lemma 9, $S_r$ must be *the* maximal exposed subset, $S^*$. $\square$

Lines 12–20 of Decomposeuse Eq. (3) to construct the good decomposition $(S,\Pi )$ of $\pi$, where $\Pi = \{\pi ^A: A \in \Psi (S)\}$. Thus, by Lemma 8, we have the following.

#### **Lemma 12**

Decompose *returns the maximal good decomposition of* $\pi$.

**Fig. 5:**
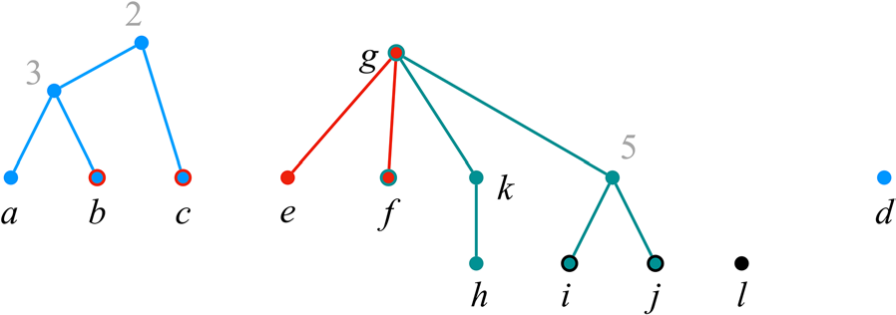
$H_{\mathcal {P}}(\pi ^\text {init}) \setminus S$. The set of exposed labels in $\pi ^\text {init}$ is $S = \{1, 4, 6\}$

Figure 5 shows the graph $H_{\mathcal {P}}(\pi ^\text {init}) \setminus S$, from which we conclude that, in line 11 of Decompose, $\Gamma = \Psi (S) = \{A_1,A_2,A_3,A_4\}$, where $A_1 = \{2, b, c\}$, $A_2 = \{d\}$, $A_3 = \{f, k, 5, g, i, j\}$, $A_4 = \{l\}$.

The **while** loop of lines 5–11 examines each label in set *S* to identify a bad label. Label 4 is bad, since $\pi _2 = \{4\}$ and $|{\text {Ch}}_{T_2}(4) \cap A_1| = |\{b,c\}| \ge 2$. Label 6 is also bad, since $\pi _4 = \{6\}$ and $|{\text {Ch}}_{T_4}(6) \cap A_3| = |\{i, j\}| \ge 2$.

Let us assume that label 6 is processed first. The body of the **while** loop replaces sets $A_3, A_4 \in \Gamma$ with their union to yield $\Gamma = \Psi (S) = \{A_1,A_2,B\}$, where $B = A_3 \cup A_4 = \{f, k, 5, g, i, j, l\}$. After this iteration, $S = \{1,4\}$.

In the next iteration, label $\ell = 4$ triggers the union of sets $A_1$ and *B*, resulting in $\Gamma = \Psi (S) = \{A_2, B'\}$, where $B'= A_1 \cup B$, and $S = \{1\}$. After this iteration, *S* contains no bad labels. Thus, by Lemma 11, *S* is the maximal nice exposed subset.

**Fig. 6:**
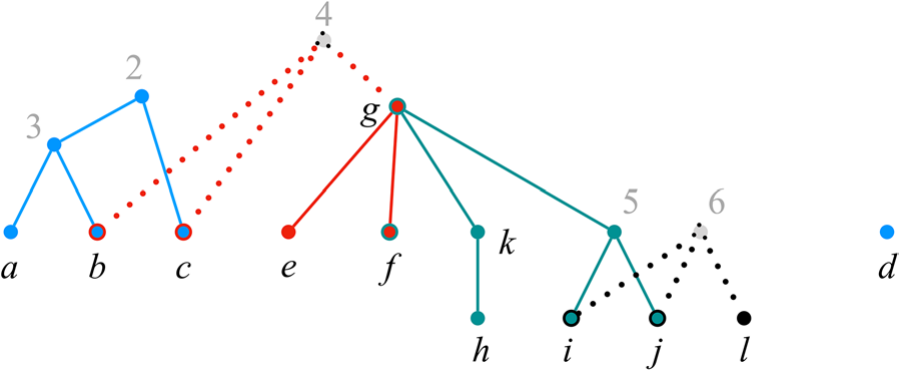
”Virtual” reconnections. The graph $H_{\mathcal {P}}(\pi ^\text {init}) \setminus S$ of Fig. 5 after virtually reconnecting labels 4 and 6

The union operations in the **while** loop can be interpreted as virtually reconnecting the bad labels—labels 4 and 6 in the example—to their children. Figure 6 uses dotted lines to represent such virtual reconnections. Each virtually connected component contains all the labels in precisely one of the sets of the collection $\Gamma$ in the minimal good partition $(S,\Gamma )$ of ${\text {Ch}}_{\mathcal {P}}(\pi ^\text {init})$. As mentioner earlier, however, for efficiency our algorithm does not actually reconnect deleted labels.

The virtually connected components are also related to the positions in the (maximal) good decomposition of ${\text {Ch}}_{\mathcal {P}}(\pi ^\text {init})$. Consider the iteration of Lines 13–20 of Decompose that processes set $A = \{2, b, c, f, k, 5, g, i, j, l\} \in \Gamma$. As explained earlier, the inner **for all** loop (lines 15–19) implements Eq. (3) to construct $\pi ^A$. The virtually reconnected labels correspond to the indices $i \in [k]$ such that $\pi ^A_i = \pi ^\text {init}_i$. In particular, iterations 2 and 4 of the inner **for all** loop set $\pi ^A_2 = \pi ^\text {init}_2 = \{4\}$ and $\pi ^A_4 = \pi ^\text {init}_4 = \{6\}$, respectively.

### Analysis

Before we analyze BuildAST’s running time, we need to specify some implementation details.

- We assume that we use the data structure of Holm et al. [15], known as *HDT*, to maintain the connected components of $H_{\mathcal {P}}$, as nodes and edges are removed from it.
- Let $\ell$ be any label in $X_{\mathcal {P}}$ and let ${\mathcal {J}}(\ell ) = \{i \in [k] : \ell \in X_i\}$. For $i \in {\mathcal {J}}(\ell )$, we say that $\ell$ is *unseen in tree* *i* if BuildAST has not yet reached a position $\pi$, such that $\ell \in \pi _i$. BuildAST maintains a list $\ell .\mathtt {unseen}$ containing all $i \in [k]$ such that $\ell$ is unseen in tree *i*. Initially, $\ell .\mathtt {unseen}= {\mathcal {J}}(\ell )$. The first time BuildAST reaches a position $\pi$ such that $\ell \in \pi _i$ for some $i \in [k]$, index *i* is removed from $\ell .\mathtt {unseen}$. Label $\ell$ is exposed when $\ell .\mathtt {unseen}= \emptyset$.
- For each $\pi$ in BuildAST’s queue, the set ${\text {Ch}}_{\mathcal {P}}(\pi )$ is stored as a sparse array $((i,{\text {Ch}}_{T_i}(\pi _i)): i \in [k] \text { and } {\text {Ch}}_{T_i}(\pi _i)) \ne \emptyset )$. This enables Decompose to access the parts of ${\text {Ch}}_{\mathcal {P}}(\pi )$ associated with each input tree separately. We use this representation of ${\text {Ch}}_{\mathcal {P}}(\pi )$ to build similar representations of the sets in the partition $\Gamma$ of ${\text {Ch}}_{\mathcal {P}}(\pi )$ produced from $H_{\mathcal {P}}(\pi ) \setminus S$ in line 11 of Decompose.
- For each label $a \in {\text {Ch}}_{{\mathcal {P}}}(\pi )$, we maintain a mapping that returns, in *O*(1) time, the set $A \in \Gamma$ containing *a*. During the execution of Decompose’s **while** loop, sets in $\Gamma$ may be merged, and representations of these merged sets must be produced and the mapping from ${\text {Ch}}_{{\mathcal {P}}}(\pi )$ to $\Gamma$ must be modified.

#### **Lemma 13**

*The total time needed to maintain the display graph throughout the entire execution of *BuildAST is ${\mathcal {O}}(nk\log ^2 (nk))$.

#### *Proof*

Initializing HDT for $H_{\mathcal {P}}$ takes ${\mathcal {O}}(nk \log (nk))$ time. Each subsequent connectivity query and edge and node deletion takes ${\mathcal {O}}(\log ^2 (nk))$ amortized time [15].

After the HDT data structure is initialized, no more edge or vertex insertions are performed. Edge deletions take place only in Line 11 of Decompose. There, $H_{\mathcal {P}}(\pi ) \setminus S$ is computed by successively deleting the edges from each label $\ell \in S$ to ${\text {Ch}}_{\mathcal {P}}(\ell )$, and then deleting $\ell$ itself. Some of these deletions may have already been performed for some ancestor position of $\pi$, where $\ell$ was also exposed. We refer to such an exposed label as *old*. Labels that are exposed for the first time in $\pi$ are *new*. We only need to delete edges from each new label $\ell$ in $\pi$, and then delete $\ell$ itself; the old labels are skipped. Therefore, each vertex and edge of $H_{\mathcal {P}}$ is deleted at most once. The total number of vertex and edge deletions over the entire execution of BuildAST is thus ${\mathcal {O}}(nk)$. The time to perform all these deletions is ${\mathcal {O}}(nk \log ^2(nk))$.

The **while** loop of lines 5–11 of Decompose merges the child sets collected in the set $\Gamma '$ constructed in Line 8. As discussed in the proof of Lemma 15, this is done without modifying the display graph. $\square$

In the following results, $d_i$ denotes the maximum number of children of a node in tree $T_i$, for each $i \in [k]$.

#### **Lemma 14**

*Excluding the time needed to maintain the display graph, Lines 2, 3, and 11 of* Decompose *take* ${\mathcal {O}}(nk \log (nk))$ *time over the entire execution of *BuildAST.

#### *Proof*

To build sets *S* and *K* in lines 2 and 3 , we do the following for each $i \in [k]$ such that $\pi _i \ne \emptyset$. Suppose $\pi _i = \{\ell \}$. If $i \in \ell .\mathtt {unseen}$, we delete *i* from $\ell .\mathtt {unseen}$. If $\ell .\mathtt {unseen}$ becomes empty, then $\ell$ is exposed. Suppose $\pi$ has a parent position $\pi ^*$. Then, exposed label $\ell \ \in \pi _i$ is *new* if $\pi _i \ne \pi ^*_i$. This step takes ${\mathcal {O}}(k)$ time per call to Decomposeand ${\mathcal {O}}(nk)$ over the entire execution of BuildAST.

To construct $\Gamma$ in line 11, we need to obtain $W \cap {\text {Ch}}_{\mathcal {P}}(\pi )$ for each connected component *W* of $H_{\mathcal {P}}(\pi ) \setminus S$. We can do this in ${\mathcal {O}}(nk\log nk)$ time, over the entire execution of BuildAST, using the technique of *scanning the smaller component*, which has been used for compatibility testing [11, 12]. Next, we outline the technique.

Let $S_\text {old}$ and $S_\text {new}$ denote the old and new labels in *S* at the beginning of an execution of Decompose; thus, $S = S_\text {old} \cup S_\text {new}$. The labels of $S_\text {old}$ and their incident edges have already been deleted. Assume that we know $W \cap {\text {Ch}}_{\mathcal {P}}(\pi )$ for each connected component *W* of $H_{\mathcal {P}}(\pi ) \setminus S_\text {old}$. We consider each node in $S_\text {new}$ in succession, deleting its incident edges one at a time. Suppose an edge deletion breaks a component *W* into two components $W_1$ and $W_2$, and assume we know $W \cap {\text {Ch}}_{\mathcal {P}}(\pi )$. We determine whether a label in $W \cap {\text {Ch}}_{\mathcal {P}}(\pi )$ ends up in $W_1$ or $W_2$ (thereby obtaining $W_1 \cap {\text {Ch}}_{\mathcal {P}}(\pi )$ and $W_2 \cap {\text {Ch}}_{\mathcal {P}}(\pi )$) as follows.

Assume without loss of generality that the smaller of $W_1$ and $W_2$ is $W_1$. We initialize $A = \emptyset$ and scan the labels of $W_1$. When we scan a label $\ell$ in $W_1$, if $\ell \in {\text {Ch}}_{\mathcal {P}}(\pi )$, we add $\ell$ to *A* and update $\ell$’s child mapping to this smaller connected component. After all edge deletions are completed, $W_1 \cap {\text {Ch}}_{\mathcal {P}}(\pi ) = A$. The set $W_2 \cap {\text {Ch}}_{\mathcal {P}}(\pi )$ consists of all labels of $W \cap {\text {Ch}}_{\mathcal {P}}(\pi )$ that were not moved to *A*. Since a label can be in a smaller component at most $\log _2 (nk)$ times and there are ${\mathcal {O}}(nk)$ labels, the total time spent in this process over all deletions performed over the entire execution of BuildASTis ${\mathcal {O}}(nk\log (nk))$. $\square$

#### **Lemma 15**

Decompose’s **while** *loop takes* ${\mathcal {O}}(k\sum _{i \in [k]} d_i)$ *time.*

#### *Proof*

By Lemma 11 the **while** loop iterates ${\mathcal {O}}(k)$ times. We complete the proof by showing that each iteration takes ${\mathcal {O}}(\sum _{i \in [k]} d_i)$ time.

Line 11 of Decomposecomputes $H_{\mathcal {P}}(\pi ) \setminus S$ by deleting at most $\sum _{i \in [k]} d_i$ edges from $H_{\mathcal {P}}(\pi )$. Therefore,

4 $$\begin{aligned} |\Gamma | \le \sum _{i \in [k]} d_i. \end{aligned}$$

For each set $A \in \Gamma$, we maintain a count, initialized to 0. By Inequality (4), the total time to initialize the counts is ${\mathcal {O}}(\sum _{i \in K} d_i)$ per iteration. To search for a bad label, for each $i \in K$, we scan each $a \in {\text {Ch}}_{T_i}(\pi _i)$, and increase the count of the set *A* to which *a* belongs. If the count for any set $A \in \Gamma$ exceeds one, then $\ell \in \pi _i$ is a bad label and the search ends.

Next, we consider the time taken by the body of the **while** loop. Retrieving $K'$ in Line 7 takes constant time. By Inequality (4) and the fact that we have constant-time access to mappings, building $\Gamma '$ in line 8 takes ${\mathcal {O}}(\sum _{i \in K'} d_i)$ time as follows. We scan each label $\ell \in {\text {Ch}}_{T_i}(\pi _i)$ for each $i \in [k]$ and retrieve the set $A \in \Gamma$ that contains $\ell$ using the mapping from ${\text {Ch}}_{\mathcal {P}}(\pi )$ to $\Gamma$. The process takes ${\mathcal {O}}(\sum _{i \in [k]} d_i)$ time per call to Decompose.

To compute the union of the sets in $\Gamma '$ in line 9, we start by initializing *B* to the empty set. We then successively consider each $A \in \Gamma '$. At each step, we append every child label $\ell$ from a non-empty entry in the representation of *A* to the corresponding entry in *B*, and change the mapping of $\ell$ to *B*. Given our representation of the sets in $\Gamma$, this process takes ${\mathcal {O}}(\sum _{i \in [k]} d_i)$ time in each iteration of the **while** loop.

Updating $\Gamma$ in Line 10 requires removing every $A \in \Gamma '$ from $\Gamma$ and then adding *B*. The time spent on updates is ${\mathcal {O}}(|\Gamma '|)$, which is ${\mathcal {O}}(\sum _{i \in K'} d_i)$. Finally, updating *S* in Line 11 takes constant time and updating *K* takes ${\mathcal {O}}(|K'|)$ time. $\square$

#### **Theorem 2**

BuildAST *can be implemented to run in *${\mathcal {O}}(n k (\sum _{i \in [k]} d_i + \log ^2(nk)))$ *time, where* *n* *is the number of distinct taxa in* ${\mathcal {P}}$, *k* *is the number of trees in* ${\mathcal {P}}$*, and* $d_i$ *is the maximum number of children of tree* $T_i$*, for* $i \in [k]$.

#### *Proof*

First, consider the total time spent on lines 2–11 of Decomposeover the entire execution of BuildAST. By Lemmas 13 and 14 , the total time spent on lines  2–11 is ${\mathcal {O}}(nk \log ^2 (nk))$. BuildASTspends ${\mathcal {O}}(nk\sum _{i \in [k]} d_i)$ time on lines 5–11 of Decomposesince, by Theorem  1, Decompose is invoked ${\mathcal {O}}(n)$ times and, by Lemma 15, each invocation spends ${\mathcal {O}}(k\sum _{i \in [k]} d_i)$ on those lines. Thus, lines 2–11 of Decomposetake ${\mathcal {O}}(nk(\sum _{i \in [k]} d_i + \log ^2(nk)))$ time over the entire execution of BuildAST.

Next, consider the **foreach** loop of lines 13–20. For each set $A \in \Gamma$ considered in that loop, Decomposeconstructs the successor position $\pi ^A$ in ${\mathcal {O}}(k)$ time. Since BuildAST generates ${\mathcal {O}}(n)$ positions, the total time spent on the loop over the entire execution of BuildAST is ${\mathcal {O}}(nk)$. This time is dominated by the time spent on lines 2–11. $\square$

## Experiments

Here we present our experimental results with a C++ implementation of BuildAST. Our source code is available on Github (https://github.com/researchGit/AgreementTesting).

As in earlier work [12], we consider two variants of BuildAST. $\texttt {BuildAST}(1)$ uses the original version of the HDT data structure, which involves *level promotion*. $\texttt {BuildAST}(0)$ uses a much simpler variant of HDT where level promotion is disallowed. (For a description of level promotion, see [15].) In [12] we showed that the simplified graph connectivity data structure outperforms the more complex data structure in the context of tree compatibility.

We performed our experiments on a machine with a 6-core i7 processor and 16 GB memory.

### Real data

We tested our program on three real profiles.

- *Spider profile:* From Figure 1 of [3]; consists of two input trees with a total of 24 labels.
- *Strepsirrhini profile:* Studied in [4]; consists of four input trees with a total of 100 labels.
- *Phocidae profile:* Studied in [3]; contains 15 input trees with 43 labels.

Our program correctly constructs an agreement tree for the Spiders profile and correctly reports that the other two profiles disagree. Since the three real profiles are small, the running times are negligible, whether we use $\texttt {BuildAST}(0)$ or $\texttt {BuildAST}(1)$.

**Fig. 7:**
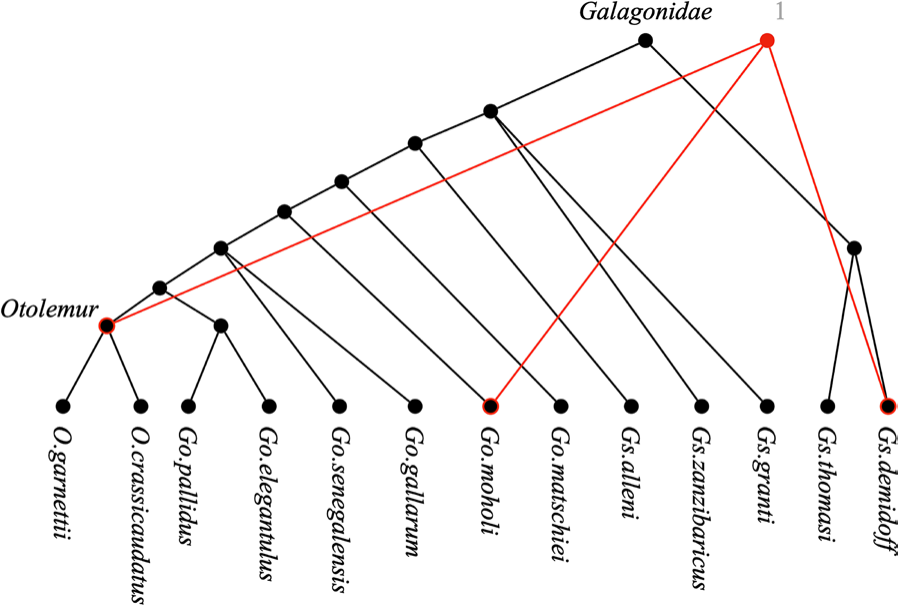
The part of display graph of the *Strepsirrhini* profile that leads to disagreement. The black tree and the red tree are subtrees of tree **b** and tree **a**, respectively, of Figure 1 in [4]

On the Phocidae profile, our program terminates immediately after processing the initial position. Indeed, the display graph of this profile has a complex structure, with several areas of disagreement. For the Strepsirrhini profile, we identified a single position of the display graph that causes disagreement. Figure 7 shows the region of the display graph corresponding to this position. The region involves taxa from two of the four input trees, colored red and black in the figure. The roots of the corresponding subtree in the black tree is *Galagoinidaea*, while for the red tree it is an internal node, originally unlabeled, to which we have assigned the artificial label 1. The conflict arises because taxa *Otolemur*, *Go. moholi*, and *Gs. demidoff* are involved in a multifurcation in the red tree, whereas in the black tree the first two taxa are contained in a subtree that does not contain the third. Because of this, after Decomposeinitializes set *S* to $\{{Galagoinidaea }, 1\}$, its **while** loop deletes both labels from *S*, leaving $S = \emptyset$.

### Simulated data

Real profiles, like those considered in the previous section, rarely agree. On such profiles, BuildAST tends to terminate quickly, without processing the input trees in their entirety. To test BuildAST’s running time on a wide range of profiles with varying numbers of taxa and trees, we devised an input generator that produces profiles that agree.

#### Experimental setup

Given integers *D* and *m*, the input generator produces a seed tree ${\mathcal {T}}^\text {seed}$ with *m* labeled nodes, where internal nodes have *D* children, and where each level except the last is completely filled. Thus, when $D = 2$, the seed tree is a complete binary tree. To generate a profile of *k* trees, we first create a collection of *k* subsets of labels, $Y_1, Y_2, \dots ,Y_k$. Each subset is obtained by choosing a random number of labels from the set of used labels (the ones chosen so far) and unused labels from the seed tree. From the collection of subsets, we produce a profile ${\mathcal {P}}= \{{\mathcal {T}}^\text {seed}|A_1, {\mathcal {T}}^\text {seed}|A_2, \dots , {\mathcal {T}}^\text {seed}|A_k\}$. Note that ${\mathcal {T}}^\text {seed}|A_i$ may contain unlabeled nodes, because certain labels in $A_i$ may have a lowest common ancestor in ${\mathcal {T}}^\text {seed}$ that is not in $A_i$. We assign such nodes new labels; by Lemma 2, this does not affect agreement.

The reported times are the averages over 30 trials. Times are given in seconds and plotted as a function of $M_{\mathcal {P}}$, the product of the number of taxa and the number of trees; i.e., $M_{\mathcal {P}}= n \cdot k$. Unless stated otherwise, the times reported are for $\texttt {BuildAST}(0)$.

#### Experiment 1: Fixed number of input trees

In the first set of experiments, we fix the number of input trees at $k = 100$. Since it is difficult to control *n*, the number of taxa, we instead vary the number of labels *m* in the seed trees from 100 to 1000 with increments of 100. The number of taxa *n* falls within a range that depends on *D*. We consider $D = 2, 3$ and 10; the respective ranges of *n* are [1135, 10875], [922, 8701] and [517, 5491].

**Fig. 8:**
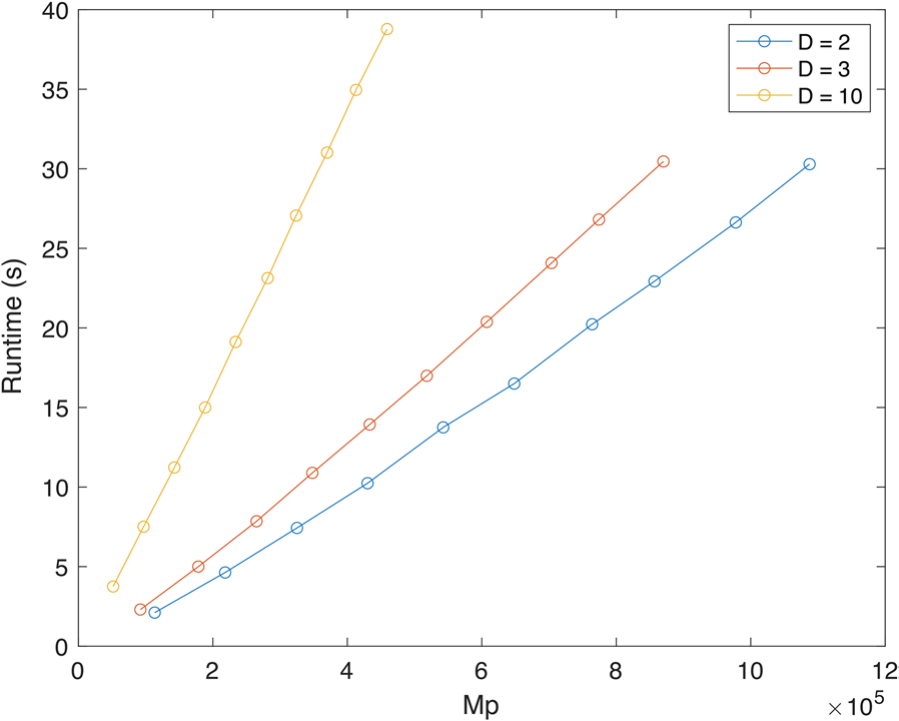
Running times for trees of degree $D = 2, 3, 10$ in profiles with $k = 100$ input trees

Figure 8 shows our results for $D \in \{2,3,10\}$. In all cases, the running time appears to be nearly linear in the number of taxa. This is partly because $\sum _{i \in [k]} d_i = k \cdot D$ is fixed. Thus the term ${\mathcal {O}}(n k \sum _{i \in [k]} d_i)$ in the time bound of Theorem 2 becomes linear in *n*. In theory, then, the ${\mathcal {O}}(n k \log ^2(nk))$ dominates the running time. In practice, however, the impact of this term appears to be less significant than the worst-case bound indicates. This seems due to the fact, previously observed in [12], that maintaining dynamic graph connectivity (the source of the polylogarithmic factor) is relatively easy on display graphs.

**Fig. 9:**
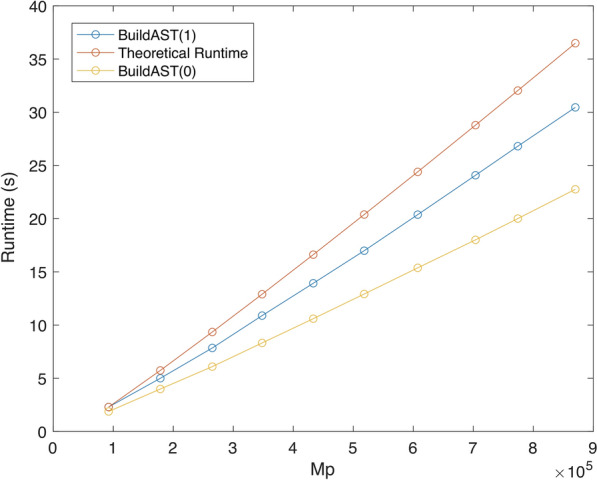
Theoretical running time versus empirical running time with and without edge promotions for $k = 100$ and trees with degree $D = 3$

Figure 9 compares the running times of $\texttt {BuildAST}(1)$ and $\texttt {BuildAST}(0)$ against the theoretical time bound for input trees with degree $D = 3$. The curves show that BuildAST performs well in practice and that $\texttt {BuildAST}(0)$ outperforms $\texttt {BuildAST}(1)$. The latter observation is similar to what we noted in [12].

#### Experiment 2: Varying the number of input trees

In the second set of experiments, we varied the number of input trees *k* from 20 to 200 with increments of 20, while keeping the number of taxa in the seed trees fixed at $m = 500$.

**Fig. 10:**
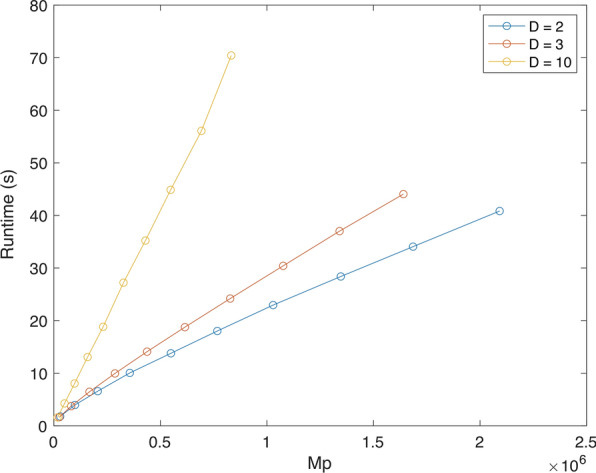
Running times for profiles of degrees 2, 3,  and 10, with *k* varying from 20 to 200

**Fig. 11:**
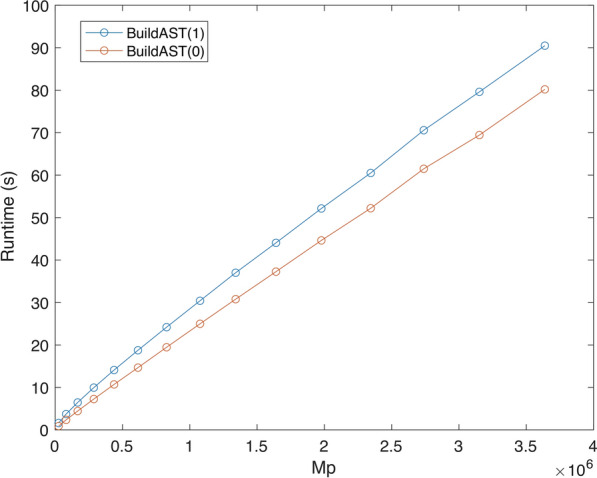
Running times with and without edge promotion for trees of degree 3, with *k* varying from 20 to 300

Figure 10 shows that when *D* equals 2 or 3, the running time grows sub-linearly at the outset, and then becomes nearly linear. In contrast, when $D = 10$, the running time curve is nearly linear. A possible explanation for these observations centers on the degree to which Decompose’s **while** loop contributes to the overall work. When $D = 10$, the input generator produces few bad labels. Thus, the **while** loop contributes little to the total time. When $D = 2$ or 3, we observe a larger number of bad labels. Since the number of trees *k* and degree *D* are small, maintaining graph connectivity initially dominates the total time, but, as the number of trees increases, the **while** loop again starts to dominate. Thus, one would expect that the running time would be closer to linear for larger numbers of trees. Figure 11, where we extend the number of input trees *k* to 300, suggests that this is indeed the case. Figure 11, also shows that, as in our first set of experiments, there is no advantage to using level promotion in HDT.

## Discussion

Theorem 2 implies that BuildAST performs well if the sum of the maximum out-degrees is small relative to the number of taxa. In fact, our experiments indicate that BuildAST is faster in practice than Theorem 2 suggests. The reason is that the proof of the theorem assumes the unlikely scenario where every edge deletion performed in constructing $H_{\mathcal {P}}(\pi ) \setminus S$ in Decompose generates a new component and that most of these components are remerged in the Decompose’s **while** loop.

The running time of BuildAST can be further improved to ${\mathcal {O}}(n k (\sum _{i \in [k]} d_i + \log ^2(nk)/\log \log (nk)))$ using the graph connectivity data structure of reference [27]. It is not clear, however, that the latter data structure is practical. In fact, the experiments we present here and in our previous work [12] suggest that data structures much simpler than HDT (and, therefore, than [27]) perform well in practice. These experimental results suggest that a more effective way to speed up BuildAST in practice would be to improve the efficiency with which Decompose deals with bad labels.

**Fig. 12:**
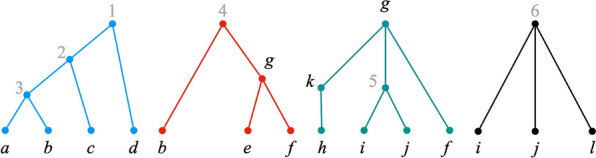
A profile ${\mathcal {P}}'= \{{\mathcal {T}}_1', {\mathcal {T}}_2', {\mathcal {T}}_3', {\mathcal {T}}_4'\}$. ${\mathcal {P}}'$ is obtained from profile ${\mathcal {P}}$ of Fig. 1 by removing taxon *c* from ${\mathcal {T}}_2$

**Fig. 13:**
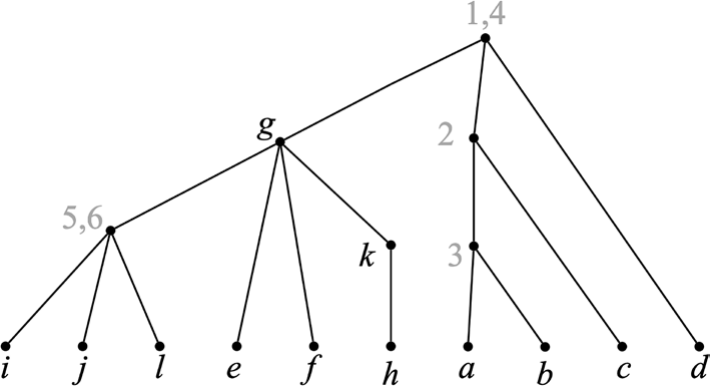
One possible agreement tree for profile ${\mathcal {P}}'$ of Fig. 12

If profile ${\mathcal {P}}$ agrees, BuildASTreturns an agreement tree ${\mathcal {T}}$ with the property that the set of labels mapped to each node in ${\mathcal {T}}$ is a maximal nice exposed subset. However, that ${\mathcal {P}}$ may have other agreement trees that do not have this property. For example, consider the profile ${\mathcal {P}}$ shown in Fig. 12. Given ${\mathcal {P}}$ as input, BuildASTreturns the agreement tree shown in Fig. 13. The tree shown in Fig. 2 (which, as we saw, is an agreement tree for the profile of Fig. 1) is also an agreement tree for ${\mathcal {P}}$, but the set of labels that map to the root of the tree is not maximal. One open question is whether it is possible to enumerate all agreement trees in time polynomial per agreement tree. A natural way to do this would be to modify Decomposeto enumerate *all* nice exposed subsets of $\pi$—not just the maximal one—efficiently. This is equivalent to Ng and Wormald’s approach to enumerating all agreement trees for a profile of leaf-labeled trees [18].

BuildAST can be modified to run in ${\mathcal {O}}(nk \log ^2 (nk))$ time for profiles ${\mathcal {P}}$ where the input trees are all binary and solely leaf-labeled. For such profiles, $|A \cap {\text {Ch}}_{T_i}(\pi _i)| \le 2$, for $A \in \Gamma$ and $i \in [k]$ in a position $\pi$ of ${\mathcal {P}}$. Labels $a, a' \in {\text {Ch}}_{T_i}(\pi _i)$ are either in the same set *A* or in different sets $A, A'$ where $A, A' \in \Gamma$. In the first case, $\ell \in \pi _i$ must be bad. Bad labels can then be detected earlier in Line 11 and directly removed from *S*. Thus, we can skip Decompose’s **while** loop. Hence, maintaining graph connectivity would dominate the performance of BuildAST.

## Conclusions

BuildAST enables users to deal with hard polytomies. In applications, we may encounter both hard and soft polytomies. It would be interesting to modify BuildAST to handle a mixture of both types polytomies, as appropriate.

## Data Availability

The code used to generate the artificial datasets analyzed in our experiments is in our Github repository. The Strepsirrhini data set was obtained from the authors of [4]. The other two real data sets are publicly available.

## Abbreviation

HDT: The dynamic graph connectivity data structure of Holm, de Lichtenberg, and Thorup [15]

## Footnotes

1 These authors refer to what we term “agreement” as “compatibility”. What we call “compatibility”, they call “weak compatibility”.
